# DG9-conjugated morpholino rescues phenotype in SMA mice by reaching the CNS via a subcutaneous administration

**DOI:** 10.1172/jci.insight.160516

**Published:** 2023-03-08

**Authors:** Tejal Aslesh, Esra Erkut, Jun Ren, Kenji Rowel Q. Lim, Stanley Woo, Susan Hatlevig, Hong M. Moulton, Simon Gosgnach, John Greer, Rika Maruyama, Toshifumi Yokota

**Affiliations:** 1 Neuroscience and Mental Health Institute,; 2 Department of Medical Genetics, and; 3 Department of Physiology, Faculty of Medicine and Dentistry, University of Alberta, Edmonton, Alberta, Canada.; 4 Department of Biomedical Sciences, Carlson College of Veterinary Medicine, Oregon State University, Corvallis, Oregon, USA.

**Keywords:** Genetics, Muscle Biology, Genetic diseases, Molecular genetics, Skeletal muscle

## Abstract

Antisense oligonucleotide–mediated (AO-mediated) therapy is a promising strategy to treat several neurological diseases, including spinal muscular atrophy (SMA). However, limited delivery to the CNS with AOs administered intravenously or subcutaneously is a major challenge. Here, we demonstrate a single subcutaneous administration of cell-penetrating peptide DG9 conjugated to an AO called phosphorodiamidate morpholino oligomer (PMO) reached the CNS and significantly prolonged the median survival compared with unconjugated PMO and R6G-PMO in a severe SMA mouse model. Treated mice exhibited substantially higher expression of full-length survival of motor neuron 2 in both the CNS and systemic tissues compared with nontreated and unmodified AO–treated mice. The treatment ameliorated the atrophic musculature and improved breathing function accompanied by improved muscle strength and innervation at the neuromuscular junction with no signs of apparent toxicity. We also demonstrated DG9-conjugated PMO localized in nuclei in the spinal cord and brain after subcutaneous injections. Our data identify DG9 peptide conjugation as a powerful way to improve the efficacy of AO-mediated splice modulation. Finally, DG9-PMO is a promising therapeutic option to treat SMA and other neurological diseases, overcoming the necessity for intrathecal injections and treating body-wide tissues without apparent toxicity.

## Graphical abstract

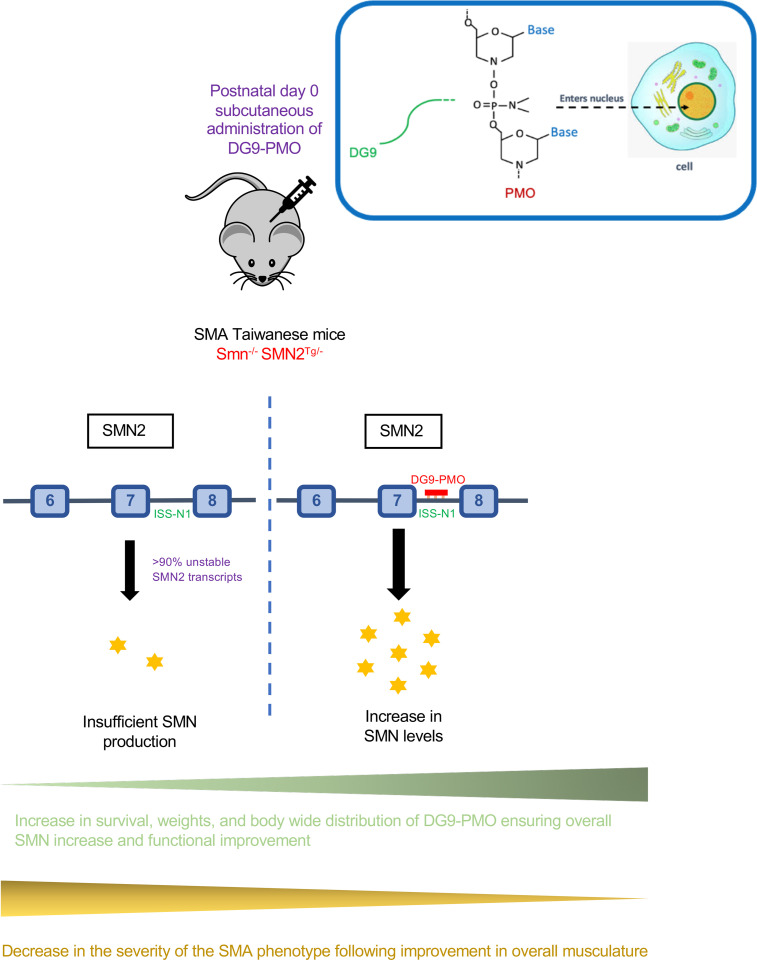

## Introduction

Spinal muscular atrophy (SMA) is a devastating neurodegenerative disorder affecting motor neurons in the anterior horn of the spinal cord (1). SMA can result in progressive muscular weakness, respiratory distress, or even paralysis and is one of the leading genetic causes of infant mortality (2). SMA is characterized by mutations in the survival of motor neuron 1, telomeric (*SMN1*), gene leading to its homozygous deletion (3). The complete loss of SMN protein is embryonically lethal (4, 5). Humans possess a paralog of *SMN1* called *SMN2*, enabling patients to be born (3, 6). However, *SMN2* cannot fully compensate for *SMN1* loss because of a C-to-T transition in exon 7, leading to its exclusion. Although the full-length *SMN2* (*FL-SMN2*) transcripts (~10% of the total *SMN2* RNAs) can produce a stable functional protein, the transcripts without exon 7 (*Δ7 SMN2*) are unstable and are rapidly degraded (~90%) (7). The exclusion of *SMN2* exon 7 is partly due to intronic splicing silencer N1 (ISS-N1) that binds to a repressor protein, heterogeneous ribonucleoprotein A1 (8–10).

All patients with SMA lack a functional *SMN1* gene, and therefore, are dependent on the *SMN2* gene to produce the SMN protein necessary for survival. The number of copies of *SMN2* is therefore a potent genetic modifier of SMA, with the copy number correlating inversely with the severity of SMA (11, 12). The inability of *SMN2* to compensate for *SMN1* loss is responsible for the preferential degeneration of motor neurons (7, 13). Although the pathological hallmark of SMA is motor neuron degeneration, recent studies highlight defects in peripheral tissues (14–16). Several in vivo mouse studies have reported bradycardia and dilated cardiomyopathy, which improved with the restoration of SMN levels (17–20).

Currently, 3 SMA-modifying treatments have been approved by the US Food and Drug Administration (FDA): nusinersen (brand name Spinraza), onasemnogene abeparvovec (brand name Zolgensma), and risdiplam (brand name Evrysdi). However, these 3 drugs have several associated concerns. Onasemnogene abeparvovec, a gene therapy drug, has been associated with hepatotoxicity and is available only for patients less than 2 years of age (21). Risdiplam is an orally deliverable small-molecule drug. However, there are safety concerns because of its associated off-target effects on pre-mRNA splicing and potential harm to embryofetal health in pregnant women, and it may also affect fertility in men (22, 23). With these drawbacks in mind, antisense oligonucleotide–mediated (AO-mediated) therapy continues to be explored for SMA. Nusinersen, a splice-switching AO with a 2′-O-(2-Methoxyethyl) (MOE) phosphorothioate chemistry, modulates *SMN2* splicing by targeting ISS-N1 and promotes the inclusion of exon 7. Since nusinersen does not cross the blood-brain barrier (BBB), it requires repeated intrathecal administration that ensures a robust restoration of SMN protein to the central nervous system (CNS) tissues (24). Despite providing a safe profile, nearly one-third of the treated patients suffer from complications associated with intrathecal administration, including persistent lumbar fluid leakage, thrombocytopenia, and other coagulation defects (24, 25). Scoliosis, a classical feature of patients with SMA, can make these injections rather challenging. Additionally, it remains controversial whether intrathecally administrated nusinersen can treat SMA symptoms other than the defect of motor function (26).

To overcome issues associated with intrathecal injections and achieve a systemic treatment, we and other groups have examined several AO chemistries and modifications, such as phosphorodiamidate morpholino oligomers (PMOs), locked nucleic acid, and constrained ethyl (27). The uncharged backbone, low protein binding affinity, and nuclease stability make PMO chemistry a promising candidate as several studies have shown that the administration of multiple high doses can be achieved with minimal toxicity (27). However, the main pitfall associated with PMO is low efficacy, which is tied to its rapid clearance from the bloodstream, poor uptake in tissues such as the skeletal muscle, and endosome trapping (28, 29). To improve the delivery of PMOs to the nuclei in target tissues and reduce the dose, numerous cell-penetrating peptide–conjugated (CPP-conjugated) PMOs are being studied. Some of these peptides facilitate the transport of PMOs across the BBB and thus ensure the possibility of noninvasive administration for the treatment of neuronal disorders (30–33). Hammond et al. showed that the systemic administration of Pip6a, a peptide from the family of PMO-internalizing peptides (Pips) into adult mice, increased *FL-SMN2* expression in the peripheral and CNS tissues (31). However, Pips are known to be highly toxic, and to our knowledge, no clinical trial has been planned (30).

To increase the delivery efficiency of AOs and reduce toxicity, we tested a peptide called DG9 for its effectiveness in an SMA mouse model (34). DG9 is derived from the protein transduction domain (PTD) of the human polyhomeotic 1 homolog (Hph-1) transcription factor, which promotes the cell membrane penetration of its protein cargoes in the lungs (35). PMOs conjugated to a near dimer of the PTD were very effective in killing bacteria (36). The DG9 peptide has an identical sequence as the dimer peptide (36) except with 5 of the dimer’s l-arginine residues replaced with d-arginine stereoisomer in order to increase its serum stability. Previously, we have found DG9 can deliver a PMO to the heart of zebrafish (34). Here, we demonstrate that a single subcutaneous injection of DG9-conjugated PMO (DG9-PMO) ameliorates the severe SMA phenotype in treated SMA mice and extends the life span by enhancing body-wide SMN restoration. DG9-PMO localizes and distributes in the CNS following a subcutaneous injection. These data indicate that DG9-PMO is a promising drug candidate for SMA and other neurological genetic disorders.

## Results

### Subcutaneous DG9-PMO administration enhances survival and improves motor function and muscle strength.

**Figure 1:**
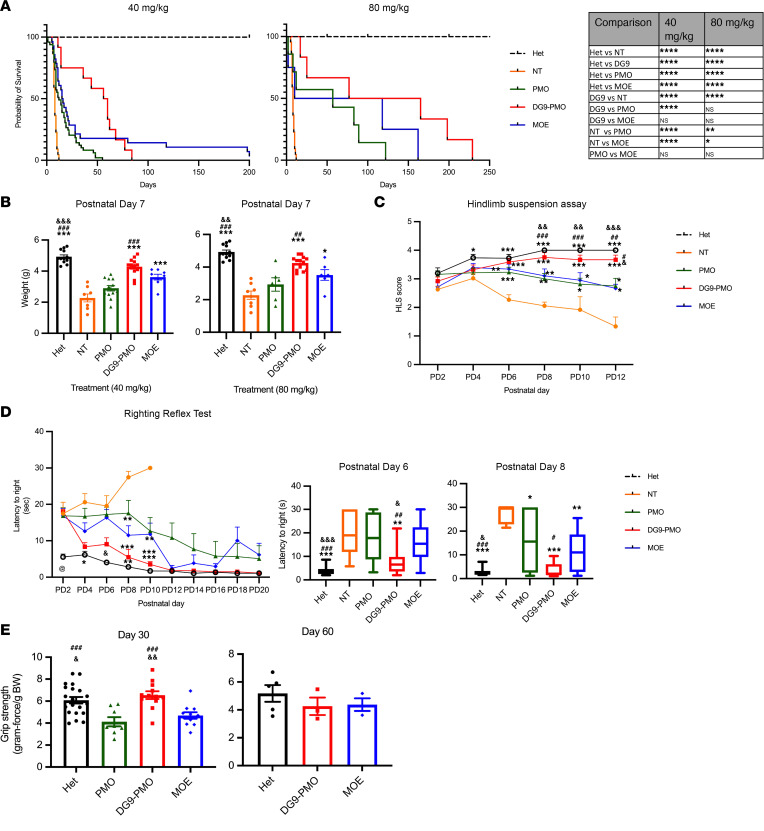
Subcutaneous administration of DG9-PMO at PD0 extends survival and improves motor function in severe SMA mice. (**A**) Survival curves of heterozygous mice (Smn^+/–^ SMN2^Tg/–^) (Hets), nontreated (NT) mice, unconjugated PMO (PMO), DG9-PMO, and MOE injected at PD0 at a dose of either 40 or 80 mg/kg. For 40 mg/kg studies, *n* = 15 (Hets), *n* = 22 (NT), *n* = 49 (unconjugated PMO), *n* = 14 (DG9-PMO), *n* = 29 (MOE). For 80 mg/kg studies, *n* = 15 (Hets), *n* = 22 (NT), *n* = 7 (unconjugated PMO), *n* = 6 (DG9-PMO), *n* = 4 (MOE) (*P < 0.05, **P < 0.01, ****P ≤ 0.0001, log-rank Mantel-Cox test). (**B**) Weight of mice at PD7 administered with either 40 or 80 mg/kg doses. Each dot (symbol) indicates a neonatal pup. (**C**) Hind limb suspension assay (HLS). Mice were treated with 40 mg/kg AOs at PD0. Score is based on the position of the hind limbs when suspended from a tube (*n* = 12–20 mice per group). (**D**) Righting reflex test. Mice were treated with 40 mg/kg AOs at PD0. The ability of mice to right themselves on their paws was measured every alternate day PD2 to PD20 (left) (*n* = 12–20 mice per group). The mean righting reflex time at PD6 and PD8 was also indicated (right: box-and-whiskers plots). Box edges, 25th and 75th percentiles; central line, median; whiskers, range. (**E**) Forelimb grip strength measured in adult mice at PD30 and PD60 from 40 mg/kg treatment groups normalized to the body weight. In **B**, **D** (box-and-whisker plots), and **E**, 1-way ANOVA followed by post hoc Tukey’s test was performed. Single symbols represent *P* < 0.05, double symbols represent *P* < 0.01, and triple symbols represent *P* < 0.005. In **C** and **D** (left graph), 2-way ANOVA followed by Holm-Šídák multiple comparison was performed. Single symbols represent *P* < 0.03; double symbols represent *P* < 0.002; triple symbols represent *P* < 0.0002. *NT, ^#^PMO, ^@^DG9-PMO, ^&^MOE. Error bars: SEM.

To examine whether DG9 peptide can increase the efficacy of PMO, we conjugated it to an 18-mer PMO with a sequence identical to nusinersen. The DG9-PMO, unconjugated PMO, and MOE were injected into SMA model mice [Cg-Tg(SMN2)2HungSmn1^tm1Hung^/J; abbreviated as Smn^–/–^ SMN2^Tg/Tg^; commonly called the Taiwanese model]. We also used an arginine-rich peptide (R6G) that is currently being explored in clinical trials for Duchenne muscular dystrophy (DMD) and used in preclinical studies for Hutchinson-Gilford progeria syndrome (37, 38). The benchmark control peptide R6G was conjugated to the same PMO sequence used in our study (R6G-PMO). We used an SMA mouse model that exhibits a severe phenotype and has a median survival of 8 days (39, 40). We subcutaneously injected mice with the AOs or saline (nontreated, NT) administered at postnatal day 0 (PD0) at 2 doses: 40 mg/kg or 80 mg/kg. In our preliminary experiments, we also tested lower doses of 10 and 20 mg/kg (data not shown). We chose higher doses of 40 and 80 mg/kg to demonstrate the safety profile and dose-dependent effect of AOs. The survival was recorded until a humane endpoint was reached. For the 40 mg/kg doses, the median survival was 12 days for unconjugated PMO, 15.5 days for MOE, and 17 days for R6G-PMO, which was increased to 58 days for DG9-PMO treatment (Figure 1A and Supplemental Figure 1A; supplemental material available online with this article; https://doi.org/10.1172/jci.insight.160516DS1). The median survival increased to 57 days for unconjugated PMO, 64 days for MOE, and 121 days for DG9-PMO at the higher dose of 80 mg/kg (Figure 1A). DG9-PMO significantly and more effectively increased the life span of SMA mice than unmodified PMO and R6G-PMO. In spite of no statistical difference between DG9-PMO and MOE groups, the median survival of DG9-PMO still was higher than MOE, as only 1 mouse was alive until PD200 following a 40 mg/kg injection of MOE.

At both doses, DG9-PMO–treated mice were significantly heavier than the NT and unconjugated PMO–treated littermates at PD7, with no significant difference when compared to the age-matched Hets that were used as healthy controls (Figure 1B). The overall appearance of DG9-PMO–treated mice looked similar to the Hets, while the unconjugated PMO–treated neonates exhibited a weak phenotype, similar to the NT mice (Supplemental Figure 1B). DG9-PMO–treated neonates also weighed significantly more than R6G-PMO–treated neonates at a dose of 40 mg/kg (Supplemental Figure 1C).

To evaluate the effects of systemic DG9-PMO treatment on muscle strength and the function of motor neurons, we used several functional tests and compared motor function in the treated and NT mice at various time points. We performed the hind limb suspension (HLS) assay and the righting reflex test during the early weeks of life. In the HLS assay, NT mice had a decreasing HLS score as they could not extend their hind limbs when suspended by the tail after PD6 (Figure 1C). These mice also exhibited a reduced latency on the tube. On the other hand, DG9-PMO–treated neonates exhibited hind limb strength comparable to the Hets at PD12, with a significantly higher score and greater latency on the tube than the unconjugated PMO–treated and NT mice (Figure 1C and Supplemental Figure 1D), indicating improvement in muscle strength. Consistent with the HLS assay, DG9-PMO mice took a significantly shorter time to right themselves on their paws between PD6 and PD10 when compared with the other groups in the righting reflex test, suggesting improved muscle strength and coordination (Figure 1D), and greater latency on the tube than the unconjugated PMO–treated and NT mice (Figure 1C and Supplemental Figure 1E). It should be noted that the results beyond PD12 are biased, as only the healthiest unconjugated PMO–, MOE-, and R6G-PMO–treated mice survived to these time points.

This improvement was further bolstered by the forelimb grip strength assessment at PD30 in the DG9-PMO–treated mice (Figure 1E and Supplemental Figure 2A). DG9-PMO–treated mice exhibited a forelimb grip force comparable to the Hets and significantly higher than the unconjugated PMO– and MOE-treated mice when injected with 40 mg/kg AOs (Figure 1E). At PD60, the performance demonstrated by the Hets, MOE-treated mice, and DG9-PMO–treated mice was similar (Figure 1E). Unconjugated PMO–treated mice did not survive until PD60 and were excluded from this portion of the study. We evaluated the relationship between the forelimb data and the sex at PD30. Surprisingly, we observed no significant difference between males across the groups but saw significant differences in the females (Supplemental Figure 2B). We also performed a rotarod test PD30 for the 40 mg/kg groups to assess their overall muscle coordination and balance (41). We observed that although the unconjugated PMO–treated mice had reduced forelimb grip strength, they were able to balance themselves on the rotating beam similar to DG9-PMO–treated mice owing to their smaller size and weight (Supplemental Figure 2C). Since we had only 2 MOE mice in this experiment, they were not included in the statistics. Taken together, based on conclusive findings from these functional tests, systemic injection of DG9-PMO improves motor function and muscle strength in both neonatal and adult SMA mice.

### Subcutaneous DG9-PMO administration increases SMN2 expression dose-dependently.

**Figure 2:**
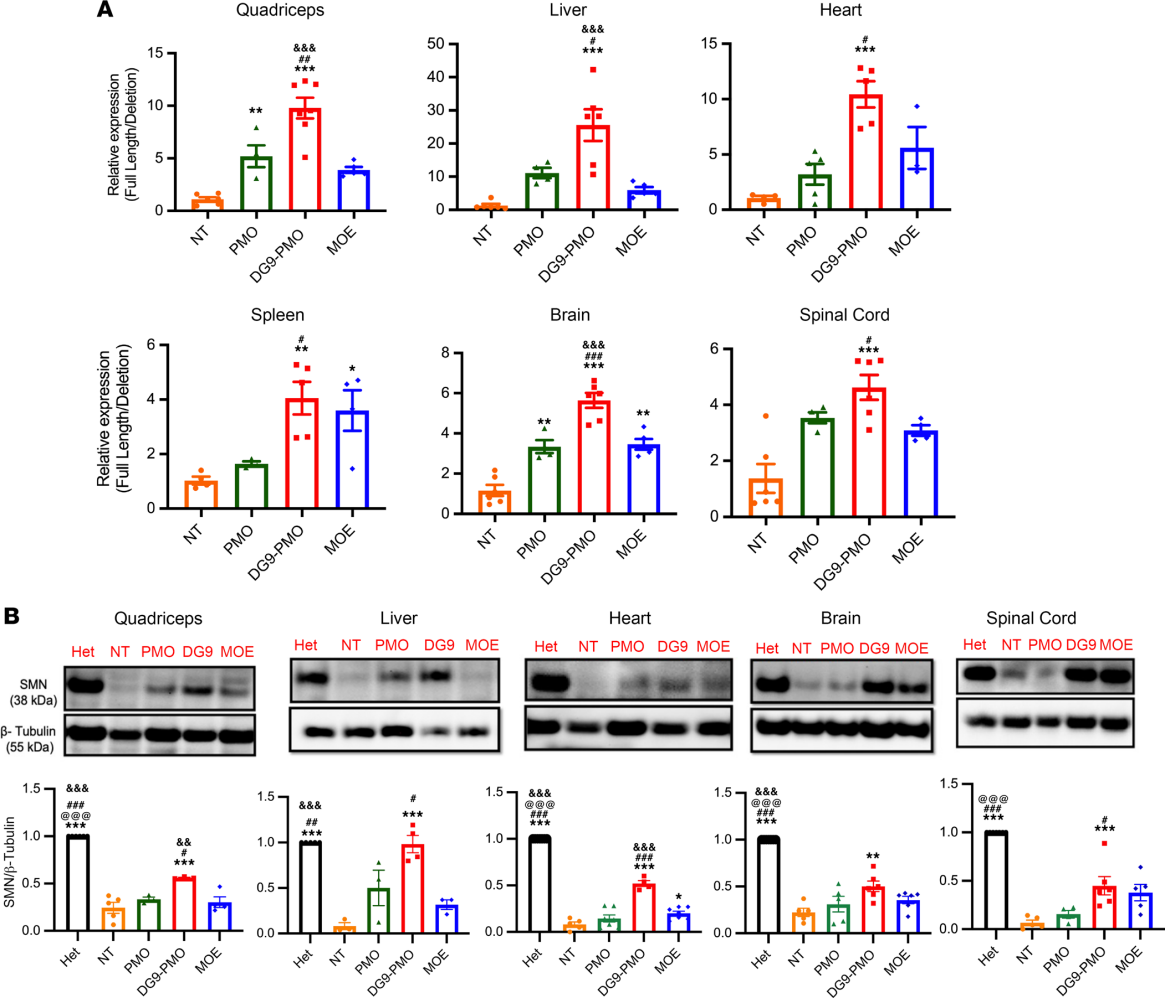
Subcutaneous administration of DG9-PMO at PD0 increases SMN expression. (**A**) Relative expression levels of full-length *SMN2* (*FL-SMN2*) compared with deleted *SMN2* transcripts (*Δ7 SMN2*) measured by quantitative PCR. (**B**) Representative images from Western blotting and the quantification of SMN protein levels, relative to β-tubulin. The Hets were used as a control with the relative SMN expression set to 1. A total of 40 mg/kg AOs were injected on PD0. Tissues were collected at PD7. One-way ANOVA followed by post hoc Tukey’s test was performed. Single symbols represent *P* < 0.05, double symbols represent *P* < 0.01, and triple symbols represent *P* < 0.005. *NT, ^#^PMO, ^@^DG9-PMO, ^&^MOE. Error bars: SEM.

To evaluate the change of *FL-SMN2* expression relative to *Δ7 SMN2* transcripts, we used real-time quantitative PCR (RT-qPCR) at PD7; the mice were treated with DG9-PMO at PD0. In both doses, DG9-PMO treatment led to a 4- to 30-fold higher *FL-SMN2* expression than NT control in both peripheral and CNS tissues (Figure 2A and Supplemental Figure 3A). It also led to a ~5-fold increase in *FL-SMN2* expression when compared with unconjugated PMO, MOE, and R6G-PMO treatments in the majority of the tissues (Figure 2A, Supplemental Figure 3A, and Supplemental Figure 1F). These data demonstrate that DG9-PMO can induce *FL-SMN2* expression more efficiently than the unmodified AOs. These findings were validated at the protein level using Western blotting, where DG9-PMO treatment increased SMN protein levels in both the peripheral and CNS tissues (Figure 2B and Supplemental Figure 3B). SMN levels were higher in CNS tissues in the DG9-PMO–treated than MOE-treated mice, though no statistical significance was seen. We also found that subcutaneous administration of DG9-PMO at 40 mg/kg led to sustained levels of SMN expression at PD30 (Supplemental Figure 4). SMN levels were similar in DG9-PMO and MOE in the tissues collected from mice at PD30.

### DG9-PMO treatment improves breathing function in neonatal SMA mice.

**Figure 3:**
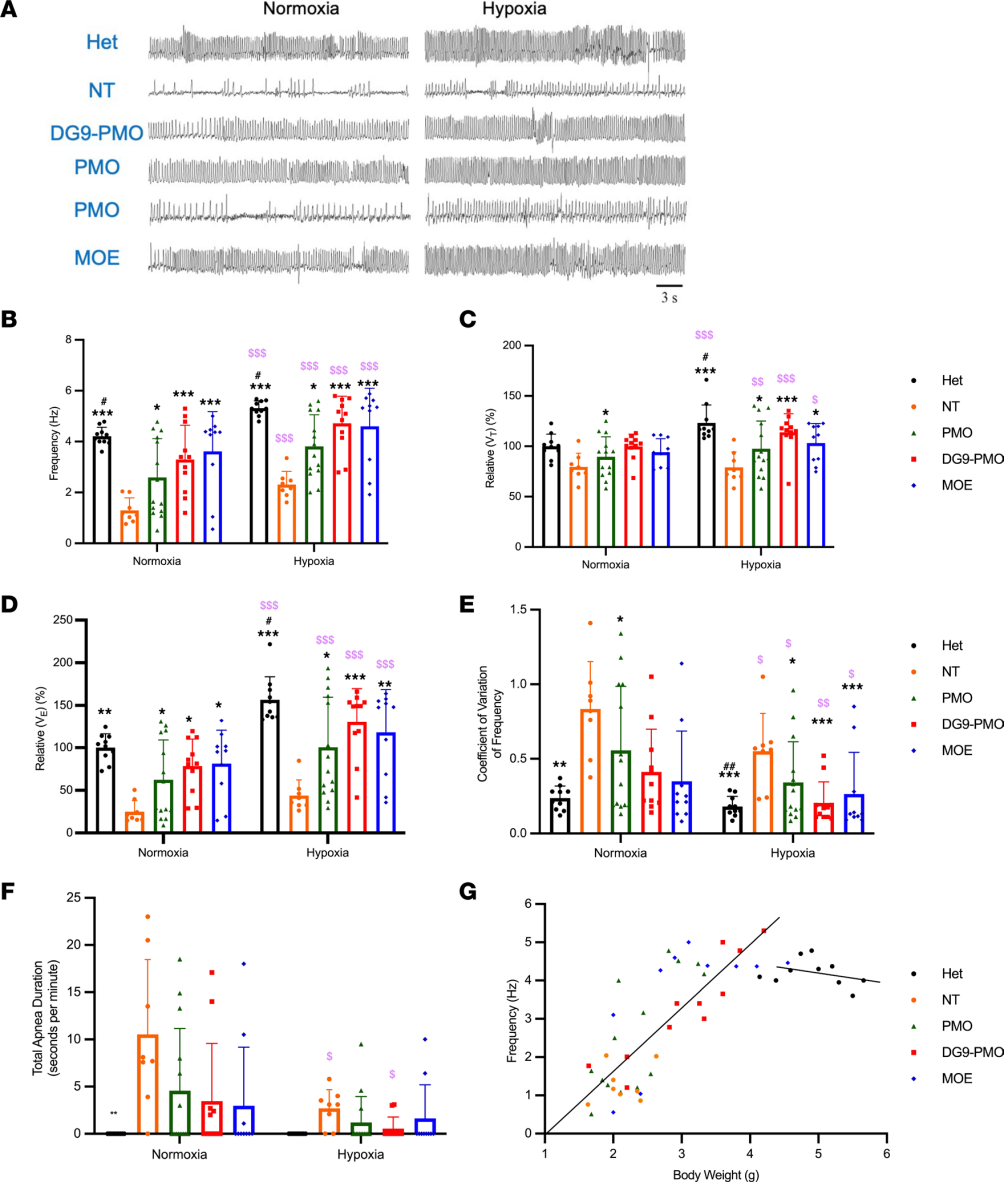
DG9-PMO treatment improves breathing function at PD7 in SMA mice. (**A**) Representative traces of whole-body plethysmograph recording from PD7 pups in normoxia (left column) and hypoxia (11% O_2_, right column) (*n* = 6 each). (**B**) Respiratory frequency (f_R_). (**C**) Tidal volume (V_T_) relative to the mean of heterozygotes (100%) in normoxia. (**D**) Minute ventilation (V_E_) relative to the mean of heterozygotes (100%) in normoxia. (**E**) Coefficient of variation of frequency (CV). (**F**) Total apnea duration (seconds in 1 minute). For those data (f_R_, V_T_, V_E_, and CV) in **B**–**E** that passed the normality test (Shapiro-Wilk) and equal variance test (Brown-Forsythe), parametric statistics were used with 2-way repeated measures ANOVA, followed by Holm-Šídák method. If the data did not pass the normality test, nonparametric statistics were used. (**G**) Correlation between respiratory frequency and body weight. Respiratory frequency was plotted against the body weight in heterozygotes (*n* = 10) with a correlation coefficient of 0.303 (*P* = 0.394). The homozygotes (*n* = 43) had a correlation coefficient of 0.791 (*P* < 0.001). A total of 40 mg/kg AOs were injected on PD0. Comparison of the difference in normoxia, or hypoxia, was conducted with Kruskal-Wallis 1-way ANOVA on ranks, followed by Dunn’s method. The difference between hypoxia and normoxia was conducted with a signed-rank test. *P* < 0.05 is taken as a statistically significant difference; single symbols represent *P* < 0.05, double symbols represent *P* < 0.01, and triple symbols represent *P* < 0.001 compared between groups indicated (*NT, ^#^PMO); ^$^ < 0.05, ^$$^ < 0.01, ^$$$^ < 0.001 compared with normoxia.

The majority of patients with SMA suffer from compromised breathing functions and rely on an external source of breathing support. To evaluate the effects of DG9-PMO treatment on breathing function, we performed whole-body plethysmography recordings at PD7 under normoxic (21% O_2_) and hypoxic (11% O_2_) conditions (Figure 3A). The Hets did not present a respiratory phenotype under normoxic conditions. The NT mice on the other hand had slow, irregular breathing denoted by a higher coefficient of variation of frequency (CV, a measure of relative variability) and marked apneas (absence of airflow/pressure changes for a period equivalent to or greater than 2 complete respiratory cycles) (Figure 3, B and F). In contrast, under the same normoxic conditions, the majority of the DG9-PMO–treated (*n* = 11) and MOE-treated (*n* = 10) mice did not exhibit any respiratory phenotype and were similar to the Hets (Figure 3, B–F). Half of the unconjugated PMO–treated mice (*n* = 14) exhibited parameters as seen in NT mice, while the other half were similar to the Hets and demonstrated an increase in f_R_ (number of breaths per minute), V_E_, and V_T_ (amount of air flowing in or out of the lungs during each respiratory cycle) when compared with the NT control (Figure 3, B and D).

When switched to a hypoxic environment, the Hets, DG9-PMO–treated mice, and MOE-treated mice exhibited an increase in the respiratory parameters f_R_, V_E_, and V_T_ relative to normoxia (Figure 3, B–D). Even though hypoxia reduced the number of apneas, breathing was still slow and irregular as seen by the high CV in NT mice (Figure 3E). Most of the unconjugated PMO–treated neonates exhibited an increase in the respiratory parameters relative to normoxia with no effects on apnea when compared to NT mice but still had slow, weak, and irregular breathing when compared with the Hets (Figure 3, B–D). We also examined the correlation of the severity of respiratory phenotype (decrease in f_R_ or V_E_) with the decrease in body weight by using the Pearson product moment correlation *t* test (Figure 3G). We observed no correlation between f_R_ and body weight in the Hets but found a strong correlation in the NT and treated mice (grouped as homozygotes). With a lower CV and a marked improvement in respiratory parameters, DG9-PMO treatment ameliorated the breathing dysfunction seen in SMA mice.

### DG9-PMO treatment improves muscle pathology and neuromuscular junction characteristics.

**Figure 4:**
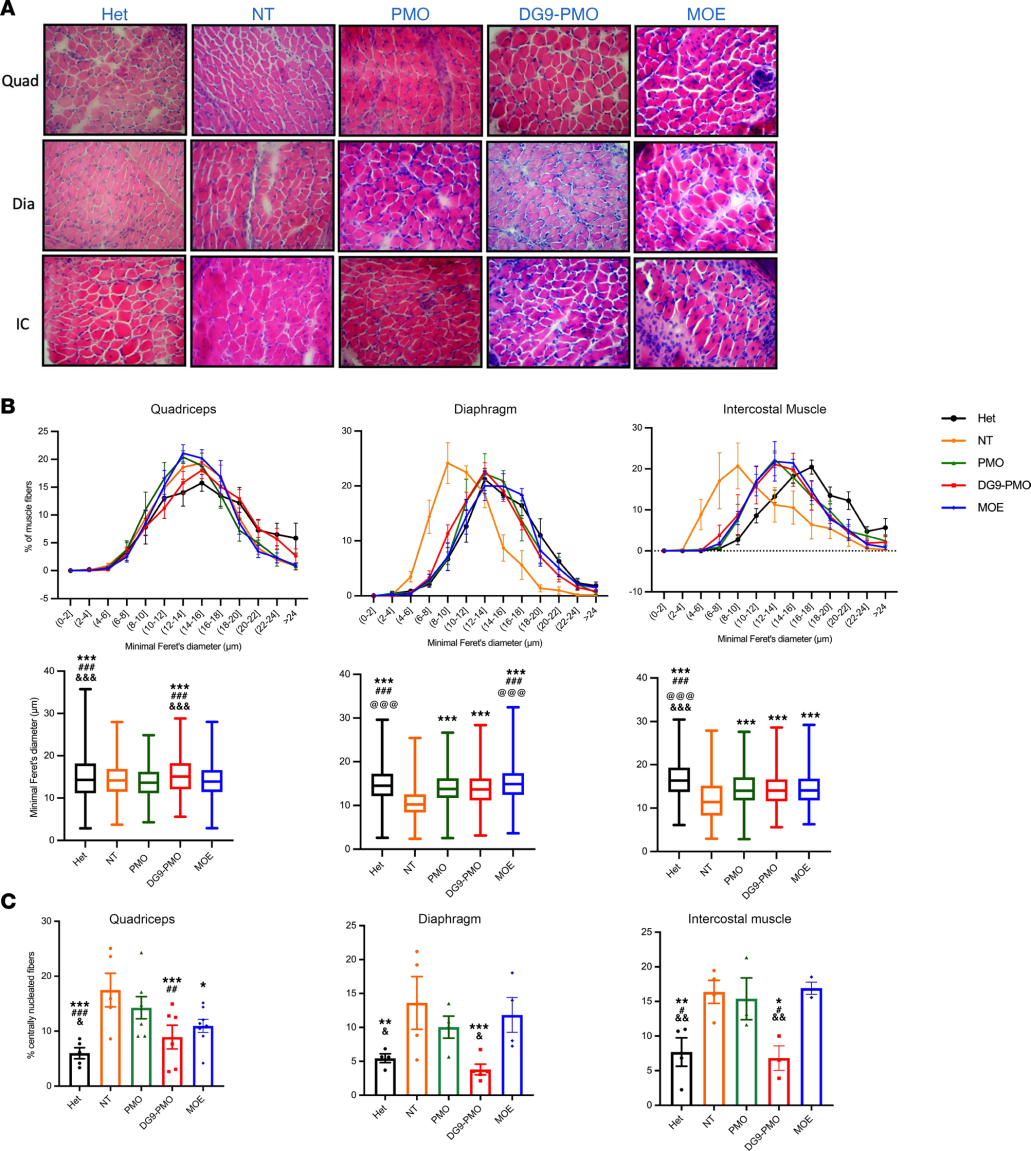
Systemic administration of DG9-PMO improves muscle pathology in SMA mice. (**A**) Representative images from H&E staining of the quadriceps muscle (top row), diaphragm (middle row), and intercostal muscle (bottom row) at PD7 in the heterozygous, NT control and treated groups. Original magnification, 40×. (**B**) Frequency distribution (top) and the quantification (bottom) of the minimal Feret’s diameter (μm) of individual myofibers. Box edges, 25th and 75th percentiles; central line, median; whiskers, range (*n* = 3–7 per group). We measured 1,292–1,653 fibers for the quadriceps, 917–1,746 fibers for the diaphragm and 642–1,127 for the intercostal muscle. (**C**) Centrally nucleated fibers quantified from the H&E images (%). A total of 40 mg/kg AOs were injected at PD0. One-way ANOVA followed by post hoc Tukey’s test. Single symbols represent *P* < 0.05, double symbols represent *P* < 0.01, and triple symbols represent *P* < 0.005. *NT, ^#^PMO, ^@^DG9-PMO, ^&^MOE. Error bars: SEM.

Atrophic musculature is a classical characteristic feature of SMA with diminished skeletal muscle fiber size. To determine the treatment effects on muscle pathology, we assessed the physiology and architecture of the myofibers of 2 affected muscle groups, the quadriceps and intercostal muscles, as well as the sparingly affected diaphragm at PD7 and PD30 (42, 43). We quantified the cross-sectional area (CSA), the minimal Feret’s diameter, and centrally nucleated fibers, a hallmark of pathological muscle degeneration/regeneration cycle, for at least 500 myofibers per muscle for each treatment using hematoxylin and eosin (H&E) staining at PD7 and PD30 (Figure 4, A and B, and Supplemental Figures 5 and 6). At PD7, the Feret’s diameter and CSA of the myofibers were significantly larger in all 3 muscle groups following DG9-PMO treatment when compared with the NT control (Figure 4B and Supplemental Figure 5). In the quadriceps muscle, DG9-PMO–treated myofiber was significantly larger than that of unconjugated PMO and MOE. In the diaphragm and intercostal muscle, all 3 treatment groups exhibited a similar myofiber size (Figure 4B and Supplemental Figure 5). In addition, DG9-PMO treatment led to a significant decrease in the percentage of centrally nucleated fibers (Figure 4C). The effect of DG9-PMO persisted at PD30 (Supplemental Figure 6). Unconjugated PMO– and MOE-treated mice had significantly smaller myofibers (Supplemental Figure 6) and a higher percentage of central nuclei (data not shown) compared with DG9-PMO in all 3 tissue types. These data demonstrate that DG9-PMOs improve muscle pathology in SMA mice.

**Figure 5:**
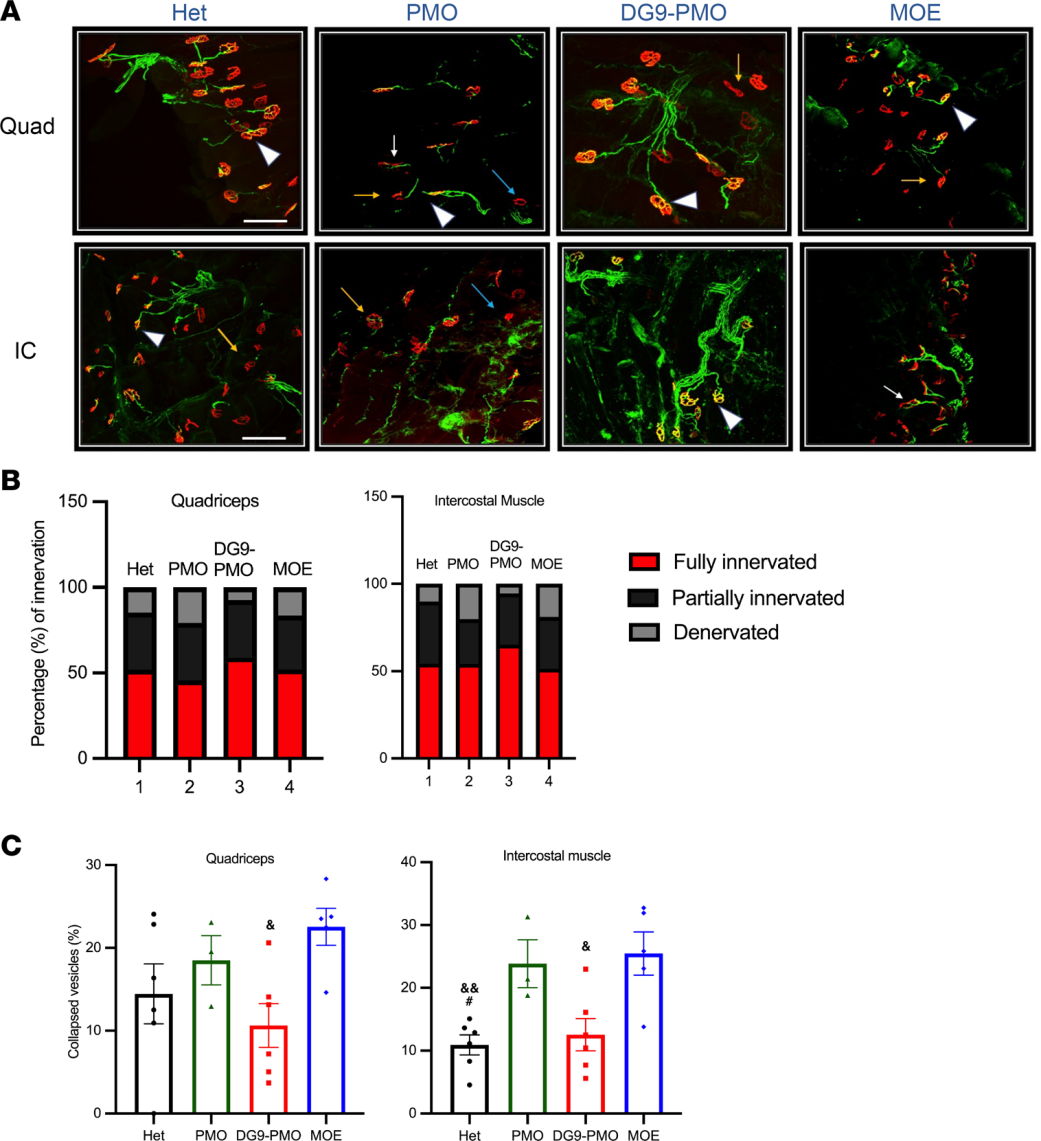
DG9-PMO treatment leads to improvement in the NMJs. (**A**) Representative confocal images of the NMJ staining in quadriceps and intercostal muscles collected at PD30. Scale bar: 100 μm. Postsynaptic endplates were stained using a-bungarotoxin (red, a-BTX) while neurofilament (2H3) and synaptic vesicles (SV2) were indicative of neurons (green). Denervated endplates can be identified as a-BTX endplates without overlapping synaptophysin-stained axons, while partially denervated endplates are identified as less than 50% occupancy of the presynaptic nerve terminals in an endplate. White arrowheads: full innervation. Yellow arrows: partial innervation. Blue arrows: denervation. White arrows: collapsed NMJs. (**B**) Innervation characteristics: full innervation, partial innervation, denervation, collapsed NMJs were quantified from at least 300–500 NMJs per group (*n* = 3–7) and plotted as percentages of total NMJs analyzed. (**C**) The number of collapsed vesicles (flat, not pretzel shaped) was quantified (*n* = 4–6 per group). Error bars: SEM. 40 mg/kg AOs were injected at PD0. In **B**, 2-way ANOVA followed by Holm-Šídák multiple comparison was used. In **C**, 1-way ANOVA followed by post hoc Tukey’s test. ^#^PMO, ^&^MOE. Single symbols represent *P* < 0.05, and double symbols represent *P* < 0.01.

The SMA mouse model typically begins to exhibit neuropathological deficits around PD4–PD5, with denervated and collapsed neuromuscular junctions (NMJs) as the disease progresses (44). We assessed the NMJ architecture of the quadriceps and the intercostal muscles at PD30 to understand the phenotypic rescue following injections at PD0 (Figure 5A). DG9-PMO treatment restored the integrity of the NMJ, increased the endplate size, reduced denervation, and exhibited innervation patterns similar to the Hets (Figure 5, A–C). The peripheral synapses in DG9-PMO–treated muscles exhibited more than 60% full innervation, while unconjugated PMO and MOE treatments had close to 40%–50% innervation (Figure 5B). Unconjugated PMO–treated mice exhibited smaller endplates and immature vesicles (termed as collapsed), especially in the intercostal muscle (Figure 5C). We also assessed the expression of atrogenes *Atrogin-1* and muscle ring finger-1 (*MuRF-1*) that are routinely upregulated in the denervated muscle (Supplemental Figure 7) (45). Although there was no statistical significance, we observed upregulation of both atrogenes in the unconjugated PMO–treated mice and upregulation of only *Atrogin-1* in MOE-treated mice in the quadriceps muscle at approximately PD30. The expression was comparable between Hets and DG9-PMO–treated mice. We also evaluated the expressions of muscle specific kinase (*MuSK*) and acetylcholine receptor alpha (*AChRα*), which play a crucial role in NMJ formation and maintenance (46–50). The expression was comparable across treatment groups for *MuSK*, while there was a reduced expression of *AChRα* in unconjugated PMO mice, albeit not statistically significant (Supplemental Figure 7). Taken together, these data indicate that DG9-PMO treatment ameliorates the phenotype of the NMJs in SMA mice. The findings corroborate our hypothesis that DG9-PMO treatment rescues the crosstalk between the muscle and the neurons by improving muscle pathology and the characteristics of NMJs.

### DG9 enhances PMO uptake in both systemic and CNS tissues.

**Figure 6:**
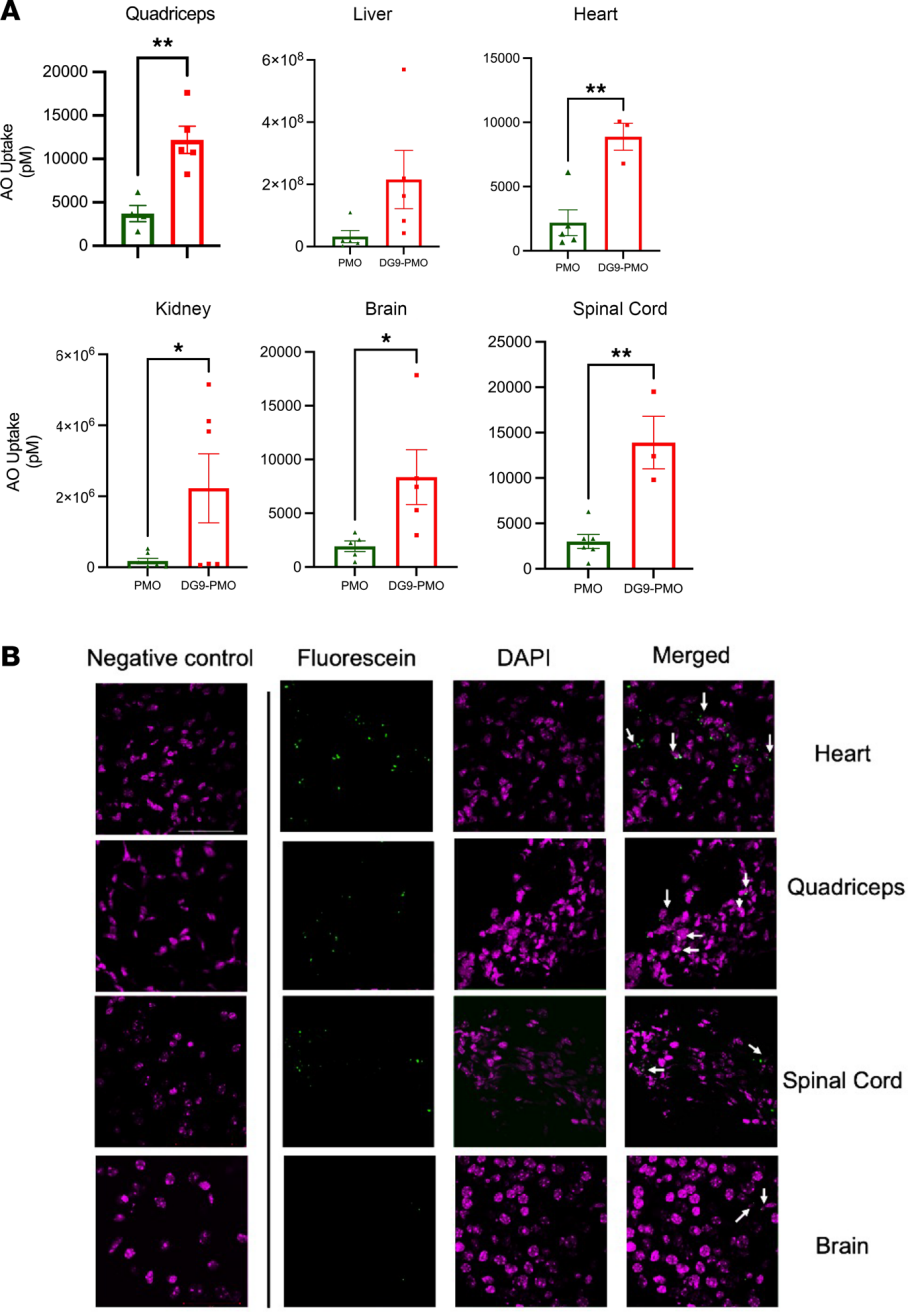
DG9 increases uptake of PMO in target tissues following subcutaneous administration at PD0. (**A**) Concentrations of PMO (pM) were measured by ELISA using the avidin-biotin affinity system and compared between DG9-PMO and unconjugated PMO treatments at 40 mg/kg doses in the quadriceps, liver, heart, kidney, brain, and spinal cord (*n* = 3–7 per group). *P < 0.05, **P < 0.01; unpaired 2-tailed Student’s *t* test. Error bars: SEM. (**B**) Representative IHC images from PD7 heart, quadriceps muscle, brain, and spinal cord following fluorescently tagged DG9-PMO subcutaneous administration at PD0. Green: fluorescein-DG9-PMO. Magenta: DAPI. DG9-PMO without fluorescent tag was used for the negative control. White arrows indicate DG9-PMO overlapped with nuclei (DAPI). *n* = 3. Scale bar: 50 μm.

To determine the effects of DG9 peptide in increasing PMO uptake, we evaluated the biodistribution of unconjugated PMO and DG9-PMO in the peripheral and CNS tissues. We used a well-established hybridization-based ELISA designed specifically to detect the PMO to compare the concentration of DG9-PMO and unconjugated PMO in the tissues of treated PD7 mice (51). Conjugation of DG9 to the PMO led to a significantly higher uptake in most tissues except for the liver, with an average AO detection of 12,202 pM (quadriceps muscle, *n* = 5), 2 × 10^8^ pM (liver, *n* = 5), 8,359 pM (brain, *n* = 5), 13,907 pM (spinal cord, *n* = 4), 8,886 pM (heart, *n* = 4), and 2 × 10^7^ pM (kidney, *n* = 6) (Figure 6A). Both unconjugated PMO and DG9-PMO displayed higher levels in the liver and kidney, as PMOs are metabolically stable and resistant to nucleases.

PMO uptake is mediated by the caveolin-dependent pathway in myotubes (52). Some CPP-PMOs get trapped in endosomes, limiting the efficiency of splicing correction (53). To reveal the intracellular localization of DG9-PMO, we attached a fluorescent tag to the DG9-PMO. We subcutaneously injected the fluorescent DG9-PMOs into SMA mice at PD0 (40 mg/kg) and performed immunohistochemistry (IHC) to examine the localization in frozen tissue sections collected at PD7. We found some DG9-PMOs localized in nuclei of cells in the hearts and quadriceps muscle and to a lesser extent in the CNS tissues (Figure 6B). This experiment shows that DG9 promotes the uptake of PMO in both the peripheral and CNS tissues following a single subcutaneous administration, thereby globally increasing SMN levels and ameliorating the SMA phenotype.

### DG9-PMO reaches the CNS in a mild SMA model.

**Figure 7:**
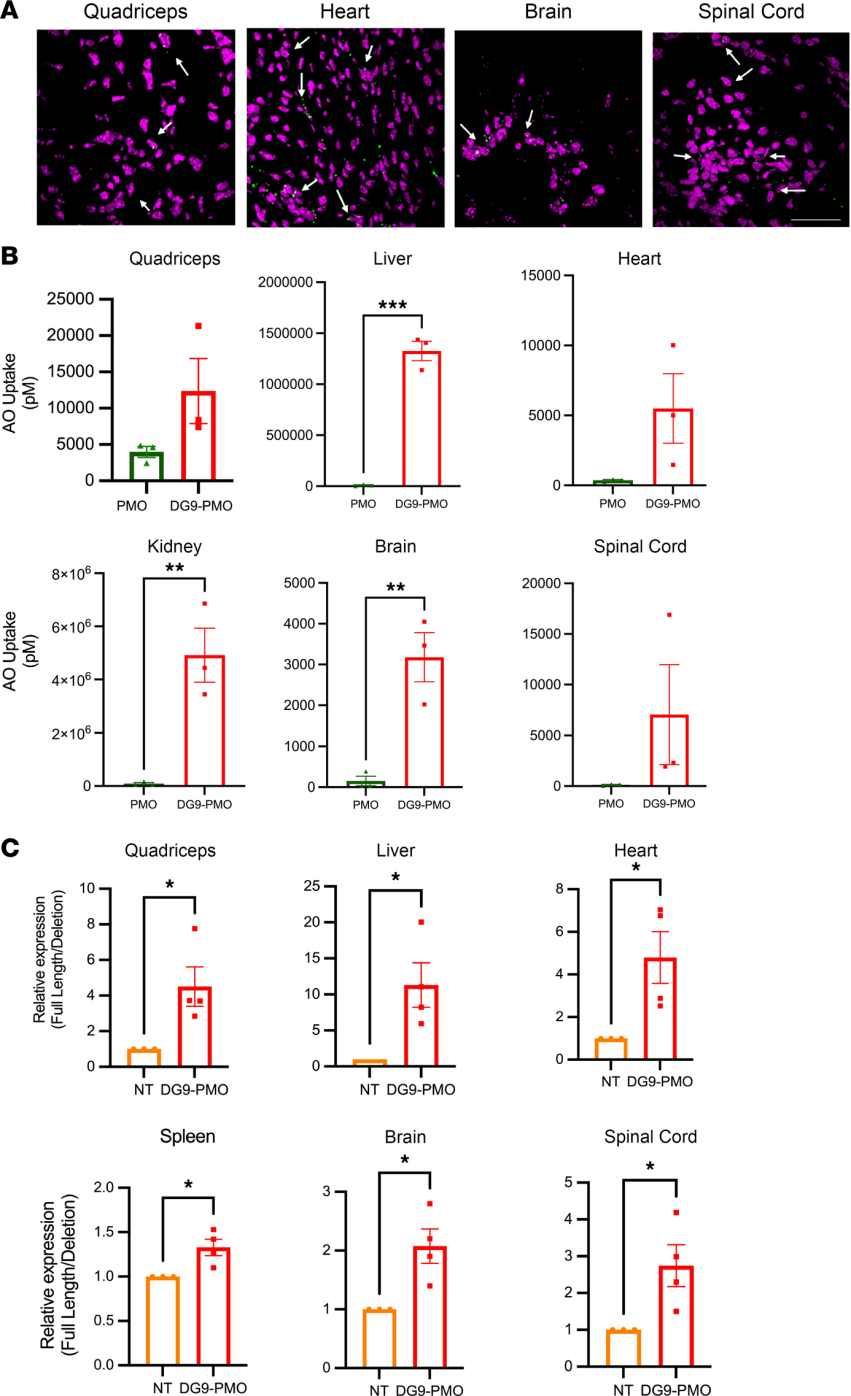
DG9-PMO reaches the CNS tissues and increases *FL-SMN2* expression in a mild SMA model. (**A**) Representative IHC images at PD7 from the quadriceps muscle, heart, brain, and spinal cord, following fluorescently tagged DG9-PMO (green) subcutaneous administration (40 mg/kg) at PD5 in the milder SMA model (F0 mice, *Smn^–/–^ SMN2^+/+^*). Magenta: DAPI. *n* = 3 per group. White arrows indicate DG9-PMO overlapped with nuclei (DAPI). Scale bar: 50 μm. (**B**) Concentrations of PMO (pM) at PD7 were detected by ELISA using the avidin-biotin affinity system and compared between DG9-PMO and unconjugated PMO treatments at 40 mg/kg injected at PD5 in the F0 mice (*n* = 3–6 per group). Statistics performed using unpaired 2-tailed Student’s *t* test. **P < 0.01, ***P < 0.001. (**C**) Relative expression levels of full-length *SMN2* (*FL-SMN2*) compared to deleted *SMN2* transcripts (*Δ7 SMN2*) in the quadriceps muscle, liver, heart, spleen, brain, and spinal cord. Saline or DG9-PMO at 80 mg/kg doses was injected at PD5 subcutaneously (*n* = 3–4 per group). The tissues were collected at PD13. In **C**, 1-way ANOVA followed by post hoc Tukey’s test was performed. *P < 0.05. Error bars: SEM.

To target the motor neurons in the CNS via systemic administration, AOs need to cross the BBB in patients with SMA. For this study, we used a milder SMA model (F0) (*Smn^–/–^ SMN2^+/+^*) with 4 *SMN2* copies obtained from Jackson Laboratory (JAX 005058) that are viable, are fertile, and have shortened, thick tails since severe SMA model mice have a median survival of only 8 days, making it difficult to evaluate the treatment efficacy when the BBB is mature. The mild model typically exhibits necrosis of the tail and ears around 8–12 weeks of age and represents type III SMA (39). To determine the ability of DG9-PMO to reach the CNS, we subcutaneously injected 40 mg/kg or 80 mg/kg fluorescently tagged DG9-PMO into F0 mice at PD0 or PD5. Fluorescently tagged DG9-PMO was detected inside the nuclei of the cells in both peripheral and CNS tissues at both PD7 and PD13 (Figure 7A and Supplemental Figure 8A), indicating that DG9-PMOs can reach the CNS tissues when the BBB is mature in SMA mice. We also performed an ELISA to examine the biodistribution of unconjugated PMO and DG9-PMO at PD7. Despite subcutaneous administration at PD5, DG9 significantly increased the uptake of PMO in the CNS and peripheral tissues at PD7 (Figure 7B). We analyzed the *FL-SMN2* levels and observed a significant increase in the *FL-SMN2* expression in both the CNS and peripheral tissues of the treated mice at PD7 (40 mg/kg) and PD13 (80 mg/kg) (Supplemental Figure 8B and Figure 7C). These findings demonstrate that the DG9-PMO can ensure widespread distribution of the AOs to both the peripheral and CNS tissues.

### DG9-PMO treatment rescues the SMA phenotype without apparent toxicity.

Peptides can typically pose as antigens, leading to immune reactions. Therefore, we examined the susceptibility of DG9-PMO to cause immune activation at an early neonatal stage, by looking at CD68^+^ cells, indicative of circulating and tissue macrophages, in the quadriceps muscle sections at PD7 (Supplemental Figure 9, A and B). The unconjugated PMO– and MOE-treated muscle had a significantly higher number of CD68^+^ macrophages when compared with the Hets, while NT and DG9-PMO mice had no significant difference (Supplemental Figure 9, A and B). The apparent reduction in circulating macrophages following DG9-PMO treatment is likely due to amelioration of the atrophic musculature, which would compensate for any elevation seen from the treatment itself.

To further elucidate the possibility of long-term toxicity, we collected serum from the Hets and AO-treated mice at PD30–PD35. A toxicological evaluation was performed on the levels of alkaline phosphatase (ALP), alanine transaminase (ALT), aspartate aminotransferase (AST), creatine kinase (CK), creatinine, total bilirubin, total protein, albumin, globulin, and gamma-glutamyl transferase (GGT). All examined indicators were comparable between the groups, suggesting no apparent toxic effects (Supplemental Figure 9C). We also performed a qualitative histological analysis of the liver and kidney and found no signs of observable toxicity (Supplemental Figure 9D). We also analyzed levels of urinary kidney injury marker-1 (KIM-1) following DG9-PMO treatment at both 40 and 80 mg/kg. We observed no apparent increase in this marker compared to the Hets at PD30 (Supplemental Figure 9E). These findings emphasize that DG9-PMO is associated with no apparent toxicity or immune dysfunction in mice.

## Discussion

In this study, we demonstrate a CPP called DG9, when conjugated to PMO, can distribute across the CNS with a single subcutaneous administration. DG9-PMO treatment significantly prolonged the survival of SMA mice and increased body weights, *FL-SMN2* expression, and SMN levels in both the peripheral and CNS tissues (Figures 1 and 2). DG9-PMO–treated SMA mice showed robust improvement in muscle strength and coordination, with significant recovery of the breathing function (Figure 3).

We employed a CPP, DG9, derived from the PTD of the human Hph-1 transcription factor, since fusion proteins containing the Hph-1 domain are known to be delivered efficiently to a wide variety of tissues, including the heart (35). We recently demonstrated successful single- and multi-exon skipping using DG9 conjugated to a different PMO cocktail targeting exons 45–55 in the dystrophin gene in vivo for the treatment of DMD (54). Similar to our DMD findings, DG9-PMO treatment resulted in higher *SMN* expression in vivo compared with unconjugated PMO and MOE (Figure 2).

Several peptide-PMO conjugates have been tested in SMA model mice to date. Hammond et al. demonstrated that Pip6a-PMOs can significantly increase the expression of *FL-SMN2* in the brain and the spinal cord of unaffected adult mice (31). Bersani et al. demonstrated that systemic injection of R6-PMOs at PD5 also increases the expression of SMN in brains and ameliorates the phenotypes of SMA *Δ*7 mice (55). Overall, these studies support that the CPP-PMO platform is a promising strategy to treat SMA systemically and overcome issues intrathecal injections cause. We compare DG9-PMO to nusinersen-equivalent MOE chemistry and a benchmark peptide-conjugated PMO (R6G-PMO) by injecting them subcutaneously at equivalent doses. R6G peptide with the glycine residue is a modification of the conventional R6 peptide that has been extensively studied for various neuromuscular disorders (32, 55, 56). Conjugation of R6G peptide shows promising results in clinical trials of DMD as well as for preclinical progeria studies (37, 38). Although R6G-PMO–treated mice exhibited an increase in *FL-SMN2* expression compared with unconjugated PMO– and MOE-treated mice, the median survival was still lower than that of DG9-PMO–treated mice (Supplemental Figure 1, A and F). One can argue about the DG9-PMO versus MOE efficacy in terms of similar SMN levels in the CNS tissues at PD30. We emphasize that peripheral restoration of SMN is also extremely vital for the overall amelioration of SMA pathology, as SMA has moved from being a “motor neuron” disease to a “whole-body” disease (57, 58). Therefore, this restoration may contribute to a greater median survival in DG9-PMO–treated mice. Over 90% of the unconjugated PMO mice were similar to the NT mice, perhaps with low SMN levels in the CNS contributing to severe motor defects. The fact that several MOE- and unconjugated PMO–treated mice died unexpectedly postnatally with only a single mouse surviving at PD200 (MOE) supports our theory that DG9-PMO can be a promising candidate to rescue the SMA phenotype.

We also demonstrated the ability of DG9 to reach the CNS by injecting a milder SMA model at PD5, when the BBB is typically mature. The proposed mechanisms for bypassing the otherwise-impermeable BBB have been discussed in reviews previously (59, 60). We chose to evaluate the efficacy approximately 7 days later, similar to our studies following AO injections at PD0. We observed localization of DG9 with enhanced uptake of PMO compared with unconjugated PMO in the CNS tissues. This provides evidence that DG9 can act as a powerful delivery vehicle to transport AOs to the CNS through noninvasive subcutaneous injections.

Although a direct comparison is not feasible because of differences in the mouse model, mode and time of administration, and so on, perhaps the advantage of DG9 is in its potentially safer toxicological profile compared with other peptides. Here, we observed no apparent toxicity at as high as 80 mg/kg dose on the liver and kidney (Supplemental Figure 9, C–E) and no morbidities or mortalities associated with toxicity following DG9-PMO treatment (Figure 1A). DG9-PMOs did not induce the increase of CD68^+^ macrophages when compared with the NT (Supplemental Figure 9, A and B). We also did not observe an upregulation in the expression of atrogenes *Atrogin-1* and *MuRF-1* following DG9-PMO treatment (Supplemental Figure 7). On the other hand, other peptide-conjugated PMOs have often induced dose-dependent toxic effects in preclinical studies and clinical trials due to the membrane-disruptive nature of CPPs associated with the amino acid compositions (29, 61). In DG9, we converted some l-arginine residues to d-arginine to improve the safety profile as previously reported (62). As opposed to most CPPs, DG9 does not contain 6-aminohexanoic acid residues either, which is known to increase toxicity (62). In contrast, previously reported peptide-PMO conjugates mostly employed l-arginine and contained multiple 6-aminohexanoic acid residues. Although these modifications might decrease the efficacy of systemic delivery, it probably improves the safety profile. Striking a balance between the efficacy and safety of peptide-conjugated PMOs (PPMOs) will be a significant focus, and a more extensive study of the pharmacokinetics and safety of PPMOs is warranted. A clinical phase II study with an arginine-rich PPMO (SRP-5051) had to be halted because of its associated toxicity in DMD following severe adverse events of hypomagnesemia (61). Recently, the FDA lifted its hold, following which the global protocol had to be adjusted to monitor urinary markers as a part of the risk assessment and mitigation strategy (63). Clinical translation of PPMOs’ delivery in patients has been challenging. Several issues need to be addressed, such as toxicity and immunogenic reactions, for example, complement activation (64). Newer Pips with fewer arginine residues have improved the toxicity profiles (53). Modifications to the chemistry, such as the addition of lipophilic conjugates such as squalene, fatty acids, and cholesterol, have improved the potency of AOs and might make them suitable to treat patients in the future (65, 66).

To our knowledge, this study is the first to demonstrate the improvement in breathing function in severe SMA mice with AO treatment. Previously, Robin et al. demonstrated improvement in respiratory outcomes in a milder type III SMA model following the administration of a tricyclo-DNA AO (67). We found that the Taiwanese SMA mouse model (Smn^–/–^ SMN2^Tg/–^) showed reduced respiratory rates and an increase in apnea frequencies at PD7 (Figure 3, A and B). Interestingly, muscle-specific SMN reduction (*MyoD-iCre SMN2^+/–^ Smn^F7/–^*) induces respiratory distress in mice, suggesting that reduction of SMN in skeletal muscles is sufficient to cause the breathing defects and the importance of SMN restoration in skeletal muscle (58). Strikingly, under both normoxic and hypoxic conditions, DG9-PMO– and MOE-treated mice exhibited significantly better respiratory outcomes than NT mice, which on the other hand, demonstrated a slow and irregular breathing pattern because of weakened atrophic muscles (Figure 3). In accordance with the result, DG9-PMO–treated muscles had larger myofiber diameters and a reduced number of central nuclei in diaphragms and intercostal muscles compared with NT mice (Figure 4). Furthermore, there was an improvement in the NMJ architecture with fewer collapsed structures and increased innervation patterns in the DG9-PMO–treated mice (Figure 5). These data support the hypothesis that DG9-PMOs rescue early respiratory dysfunction in SMA mice and highlight the necessity of systemic treatment for SMA, including skeletal muscle.

In summary, we have demonstrated the efficacy and safety of a peptide DG9-PMO conjugate that ensures widespread distribution of PMO to both the CNS and peripheral tissues in a severe SMA mouse model following a single subcutaneous administration. This led to a significant increase in survival and SMN levels, restored muscle integrity, and most importantly rescued the severe respiratory phenotype without apparent toxicity. DG9-PMO treatment is important from a clinical perspective because it can circumvent issues associated with intrathecal injections, hepatotoxicity, and nonspecific targets associated with the currently approved treatments for SMA and provides a proof of concept for the treatment of other neuronal disorders that typically face challenges and limitations due to poor uptake of AOs.

## Methods

### Synthesis of AOs used in the study.

DG9 (sequence N-YArVRRrGPRGYArVRRrGPRr-C) and R6G (sequence RRRRRRG) (uppercase: l-amino acids, lowercase: d-amino acids) (WO2019067975A1) were synthesized and covalently conjugated to the 3′ end of the PMO using the method described previously (68). The PMO targeting ISS-N1 intron 7 (5′ TCACTTTCATAATGCTGG 3′) was purchased from Gene Tools LLC. 2′MOE was purchased from Eurogentec North America.

### Mice.

SMA-transgenic mice [JAX stock 005058 FVB.Cg-Tg(SMN2)2HungSmn1^tm1Hung^/J] (Smn^–/–^; SMN2^Tg/Tg^), also known as Taiwanese mice, were purchased from Jackson Laboratory. Heterozygous mice for Smn1 (Smn1^+/–^; SMN2^–/–^) generated by us were crossed with mice homozygous for Smn1 Smn^–/–^; SMN2^Tg/Tg^ generated by us to obtain the SMA mice (Smn^–/–^; SMN2^Tg/–^) or the heterozygous healthy control (Smn1^+/–^; SMN2^Tg/–^). The SMA mice display a severe overt phenotype similar to patients with SMA type I. The mice were genotyped using a PCR assay on genomic DNA isolated from tail biopsies using the Phire Tissue Direct PCR Kit (Thermo Fisher Scientific) as per the manufacturer’s instructions. The DNA was amplified using the primers described in Supplemental Table 1 using the condition Mouse Smn: 98°C/5 minutes *→* 98°C/5 s 58°C/10 s 72°C/10 s × 35 cycles → 72°C/1 minute.

### Treatment.

Injections were carried out using a 30-gauge Hamilton syringe. SMA mice identified following genotyping were randomly assigned to a treatment group. The researcher injecting mice was blinded to the treatment. SMA neonates were subcutaneously injected with 40 or 80 mg/kg of AOs at PD0, while NT and heterozygous control mice were injected with saline (*n* = 10–49) per group. Tissues including the quadriceps muscle, liver, kidney, spleen, diaphragm, intercostal muscle, heart, brain, and spinal cord were collected and snap-frozen in dry ice– cooled isopentane, then subsequently stored at –80°C.

### RT-qPCR.

Frozen tissues harvested at PD7 were sectioned using a cryostat (Leica CM 1950) into 20 μm sections. RNA was extracted from these sections using TRIzol Reagent (Invitrogen). cDNA was synthesized from 50 ng/μL RNA using SuperScript IV Reverse Transcriptase (Thermo Fisher Scientific) and oligo(dT) primers (Thermo Fisher Scientific) following the manufacturer’s instructions. qPCR reaction was performed using the SsoAdvanced Universal SYBR Green Supermix (Bio-Rad) and QuantStudio3 real-time PCR system (Applied Biosystems). The primers are included in Supplemental Table 1. The relative gene expression for *FL-SMN2* over the deletion of SMN2 transcripts without exon 7 was normalized to the NT control samples and analyzed using the ΔΔCt method. The expression of denervation markers relative to 18S was normalized to the healthy heterozygous control samples collected at PD30 and analyzed using the ΔΔCt method.

### Western blotting.

Total protein was extracted from frozen tissues harvested at PD7 or PD30 by using RIPA buffer (MilliporeSigma) with cOmplete, Mini, EDTA-free protease inhibitor cocktail (MilliporeSigma). The protein concentration was quantified using the Pierce BCA Protein Assay Kit (Thermo Fisher Scientific). For SDS-PAGE, 5–10 μg of protein per well was run in NuPAGE Novex 4%–12% Bis-Tris Midi protein gels (Life Technologies) at 150 V for 60 minutes, followed by semidry transfer at 20 V for 30 minutes. The polyvinylidene fluoride membrane was blocked overnight with 5% skim milk and 0.05% Tween 20 in PBS (PBST). The membrane was incubated with a mouse purified anti-SMN antibody (610647, BD Biosciences) (1:10,000) for 1 hour at room temperature (RT) under agitation. The membrane was washed 3 times with PBST (10 minutes each wash) and incubated with HRP-conjugated goat anti-mouse (IgG H+L; 31430, Invitrogen) at RT for 1 hour under agitation. The bands were detected using the Amersham ECL Select Western Blotting Detection Kit (GE Healthcare) and visualized by the ChemiDoc Touch Imaging system (Bio-Rad). For β-tubulin, the membrane was incubated with the stripping buffer (15 g glycine, 1 g SDS, 10 mL Tween 20 at pH 2.2) for 10 minutes at RT, followed by 2 washes in PBS (10 minutes each) and 2 washes in Tris-buffered saline and 0.05% Tween 20 (5 minutes each). Similar to the primary antibody protocol, the membrane was subsequently blocked overnight and incubated with β-tubulin rabbit antibody (Abcam ab6046, 1:5,000) at RT for 1 hour under agitation the next day. The secondary antibody used was HRP-conjugated goat anti-rabbit (IgG H+L; 31460, Invitrogen) for Tubulin (Bio-Rad, 1:10,000). The bands were visualized as mentioned previously. See complete unedited blots in the supplemental material.

### ELISA.

ELISA was performed as described previously (51, 69). In brief, protein was extracted from frozen tissue sections (~20 μm) using RIPA buffer with cOmplete, Mini, EDTA-free protease inhibitor cocktail. The probes (Integrated DNA Technologies) were designed complementary to the PMO sequence with phosphorothioated backbones at the 5′ and 3′ ends. The 5′ and 3′ ends were labeled with digoxigenin and biotin, respectively. The tissue lysates (0.02 mg/mL protein concentration) were pretreated with 2.5 mg/mL trypsin containing 10 mM CaCl_2_ at 37°C overnight to digest the DG9 peptide. The probes were added to the samples and allowed to hybridize at 37°C for 30 minutes. Following the probe-PMO hybridization, the hybridized mixtures were added to Pierce NeutrAvidin Coated 96-Well Plates, Black (Thermo Fisher Scientific), to allow avidin-biotin interaction between the plate and the probes. The unhybridized probes were digested using micrococcal nuclease enzyme at 0.1 gel unit/μL (New England Biolabs). This was followed by the addition of an anti-digoxigenin antibody conjugated with ALP 1:5,000 (11093274910, Roche Applied Sciences). Attophos AP Fluorescent Substrate (Promega) was added to the PMO/DG9-PMO probes, and fluorescence was detected at 444 nm excitation and 555 nm emission by using a monochromator SpectraMax M3 plate reader (Molecular Devices).

### H&E staining.

Cryosections (7 μm) of the quadriceps muscle, diaphragm, and intercostal muscle were stained with Meyer’s H&E reagents (Electron Microscopy Reagents) (70). For the centrally nucleated fibers and myofiber size, 500–800 were randomly selected per muscle per treatment. The CSA and minimal Feret’s diameter were quantified using ImageJ (NIH). All H&E analyses were performed in a blinded fashion.

### NMJ staining.

NMJ staining was carried out as previously described (71). Briefly, the quadriceps and the intercostal muscle were fixed in 4 % paraformaldehyde (PFA) for 2 hours. The muscles were washed quickly with PBS and blocked with 5% goat serum and 2 % Triton-X in PBS for 1 hour at RT. This was followed by overnight incubation at 4°C with the primary mouse monoclonal antibodies anti-neurofilament clone 2H3 (1:100) and anti-synaptophysin clone SV2 (1:500) (DSHB). Subsequently, the muscles were incubated in the dark with Alexa Fluor 488–goat anti-mouse IgG1 (1:1,000) (Life Technologies) and Alexa Fluor 594–bungarotoxin (1:5,000) (Thermo Fisher Scientific) (A21121 and B13423, respectively). At least 300 NMJs were *Z*-stacked and visualized for the analysis of denervation and CSA using a confocal microscope (Zeiss LSM 710) and Zeiss ZEN software. The synaptic area was quantified using ImageJ. All analyses were performed in a blinded fashion.

### Macrophage detection.

Cryosections (7 μm) of the quadriceps muscle were fixed with 4% PFA. The sections were incubated with rat anti-mouse CD68 antibody (MCA1957T, Bio-Rad). The number of CD68^+^ cells was counted and averaged from at least 5 sections per sample at random intervals at 20× original magnification.

### Righting reflex test.

This spontaneous ability of the mice to right themselves up was tested from PD2 until the weaning stage PD20. The neonates were placed on their backs, and the time taken to reposition and place all 4 paws on the ground was noted. The recording time was a maximum of 60 seconds. Each neonate underwent 3 trials, with a resting period of at least 10 minutes between trials. The data are expressed as the mean time to complete the righting reflex test ± SEM.

### HLS assay (tube test).

This test was performed on neonatal mice PD2–PD12 as described in Treat NMD protocol SMA_M.2.2.001 (72). Briefly, neonatal mice were suspended on their hind limbs from a tube. They were scored based on the position of the hind limbs and their latency to fall was recorded with a 30-second cutoff. Each neonatal pup underwent 3 trials, with a 15-minute break between each trial. The average score was noted by the observer under a blinded test protocol.

### Forelimb grip strength.

This assay was conducted as described in Treat NMD protocol SMA_M.2.1.002 (73). In short, the mouse is placed on the wire mesh of an automated grip strength meter (Columbus Instruments) such that only the front paws are allowed to grip the metal grid. The mouse is steadily pulled with the help of its tail, such that it lets go of the grid completely. PD30 and PD60 mice were used. Each mouse underwent 3 trials.

### Rotarod test.

The rotarod test (AccuScan Instruments) was performed on mice between PD30 and PD40, using an acceleration profile of 300 seconds as previously described (74).

### Toxicology.

Blood was collected from the mice around PD30–PD40 (*n* = 4–10 per group) during the dissection procedure. The blood was left at RT for 30 minutes and centrifuged at 2,000 rcf. The resulting supernatant (serum) was transferred to a new 1.5 mL tube and stored at –20 °C. The serum samples were analyzed by IDEXX BioAnalytics. The evaluation was performed on a standard set of toxicity markers: glucose, total bilirubin, blood urea nitrogen, CK, creatinine, ALP, ALT, AST, GGT, globulin, albumin, and total protein. Urinary KIM-1 analysis was performed from urine collected from mice at PD30. The collected urine was centrifuged at 2,000 rcf at 4°C. The resulting supernatant was transferred to a new 1.5 mL tube and stored at –20°C until further use. Analysis was performed using a single-wash 90-minute sandwich ELISA protocol according to the manufacturer’s instructions (Abcam ab213477).

### Whole-body plethysmographic recordings.

Measurements were performed in whole-body, cylindrical, transparent, plexiglass plethysmographs (Validyne Engineering) that had 1 inflow and 2 outflow ports for the continuous delivery of fresh room air and removal of expired carbon dioxide (75, 76). The plethysmograph of volumes was 10 mL (inner diameter: 1.9 cm, length: 3.5 cm for PD7 mice with body weight less than 2.5 g), 30 mL (inner diameter: 2.6 cm, length: 5.6 cm for PD7 mice with body weight more than 2.5 g), and 80 mL (inner diameter: 3.8 cm, length: 7 cm for PD30) for measures of respiratory parameters with a flow rate of 15, 45, and 120 mL/min, respectively. The gas was mixed with gas mixer (GSM-3, CWE), delivered from compressed pure oxygen and pure nitrogen cannisters, being monitored using 0–200 mL/min gas regulators (Porter Instrument Company). Hypoxic challenge (11% of oxygen for 5 minutes) was performed with continuous monitoring of plethysmographic recordings without physical handling of the animal by switching inflow gas from normoxia (21% of oxygen, balanced by nitrogen) to hypoxia (11% of oxygen, balanced by nitrogen). It took about 1 minute to finish gas exchange, confirmed with gas analyzer (ML206, ADInstruments). For PD7, the plethysmograph was contained within an infant incubator (Isolette, model C-86; Air-Shields/Dräger Medical) to maintain the ambient temperature at the approximate nest temperature of 32°C. For PD30, the plethysmograph was recorded at RT of around 22°C. Pressure changes were detected with a pressure transducer (DP 103; Validyne) and signal conditioner (CD-15; Validyne), recorded with data acquisition software (Axoscope) via analog-digital board (Digidata 1322A). Signals were high-pass–filtered (0.01 kHz), with a sampling rate at 1 kHz. V_T_ and f_R_ were measured with blood pressure settings using Labchart 8 (ADInstruments). Threshold levels for bursts were set, and bursts were then automatically detected so that frequency (calculated from cycle duration) and V_T_ (calculated from maximal pressure minus minimal pressure) values were interpreted.

It should be noted that our plethysmograph is effective for studying f_R_ and detection of apneas. An apnea is defined as the absence of airflow (pressure changes) for a period equivalent to or greater than 2 complete respiratory cycles. Our whole-body plethysmographic system provided semiquantitative measurements of V_T_ (mL/g) and minute ventilation (V_E_ = f_R_ × V_T_: mL/min/g), from which we report changes relative to the wild-type normoxia (75, 76). The CV, a measure of relative variability, is the ratio of the standard deviation to the mean (average). The smaller the ratio, the more regular the breathing. The experiments were conducted between 10 am and 5 pm. Animals were sent back to the animal facility after experiments.

Respiratory parameters were calculated over an average of 1 minute of continuous plethysmography recordings. The respiratory parameters V_T_ and V_E_ were reported relative to the mean of heterozygotes in normoxia (100%). The nature of the hypothesis testing is 2 tailed. We first ran the normality test (Shapiro-Wilk) and equal variance test (Brown-Forsythe). For those data (Figure 3, B–E: f_R_, V_T_, V_E_, and CV) that passed both tests, parametric statistics were used with 2-way repeated measures ANOVA, followed by Holm-Šídák method (2 factors: different treatments and different conditions). *P* < 0.05 is taken as a statistically significant difference; *n* refers to the number of animals, with animal as the unit of analysis for statistical tests. For those data (total apnea duration, Figure 3F) that failed either the normality test or equal variance test, nonparametric statistics were applied. Comparison of the difference in normoxia or hypoxia was conducted with Kruskal-Wallis 1-way ANOVA on ranks, followed by Dunn’s method. The difference between hypoxia and normoxia was conducted with a signed-rank test. To examine if severity of respiratory phenotype (decrease of f_R_ or V_E_) was correlated with decrease of body weight, we used the Pearson product moment correlation *t* test (Figure 3G). For whole-body plethysmography recordings, data are expressed as mean ± SD, or first interquartile 25%, median 50%, and third interquartile 75% (Sigmaplot 11 Systat Software Inc.).

### Statistics.

All statistical analyses for all data (except respiratory analysis) were performed with GraphPad Prism 9 software. One-way ANOVA with Tukey’s test for multiple comparisons, or log-rank Mantel-Cox (*P* ≤ 0.0001) test for survival analysis, was used as needed. *P* < 0.05 was considered significant for 1-way ANOVA studies. For righting reflex test, HLS assay, and NMJ innervation statistics, 2-way ANOVA with Holm-Šídák multiple-comparison test was performed and *P* < 0.03 was considered significant. For ELISA studies, unpaired 2-tailed Student’s *t* test was used and *P* < 0.05 considered significant. No statistical power calculation was conducted before the study.

### Study approval.

All animal experiments were conducted at the University of Alberta and approved by the Animal Care and Use Committee, University of Alberta Research Ethics Office.

## Author contributions

TA, RM, SG, JG, and TY designed the research. HMM and SH designed the peptide DG9 and conjugated it to the PMO. TA, EE, and JR performed experiments. EE, SW, and KRQL performed blinded analysis. TA, JR, RM, and TY wrote the manuscript.

## Supplementary Material

Supplemental data

## Version 1 01/31/2023

In-Press Preview

## Version 2 03/08/2023

Electronic publication
