# Supplementary material for: DG9-conjugated morpholino rescues phenotype in SMA mice by reaching the CNS via a subcutaneous administration
Source: JCI Insight. 2023 Mar 8;8(5):e160516. doi: 10.1172/jci.insight.160516 (PMC10077475; doi:10.1172/jci.insight.160516)

Supplemental Figures

Figure 1.

A

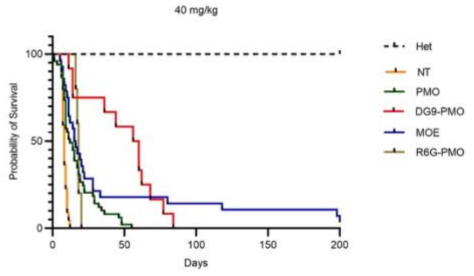

Comparison	40 mg/kg	80 mg/kg
NT vs R6G-PMO	****	NA
MOE vs R6G-PMO	ns	NA
DG9 vs R6G-PMO	*	NA
PMO vs R6G-PMO	ns	NA
Het vs R6G-PMO	***	NA

B

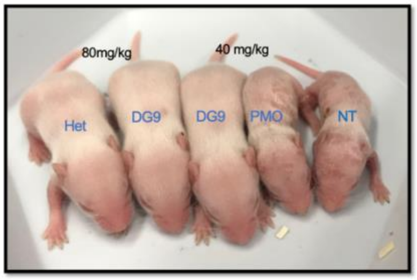

C D E F

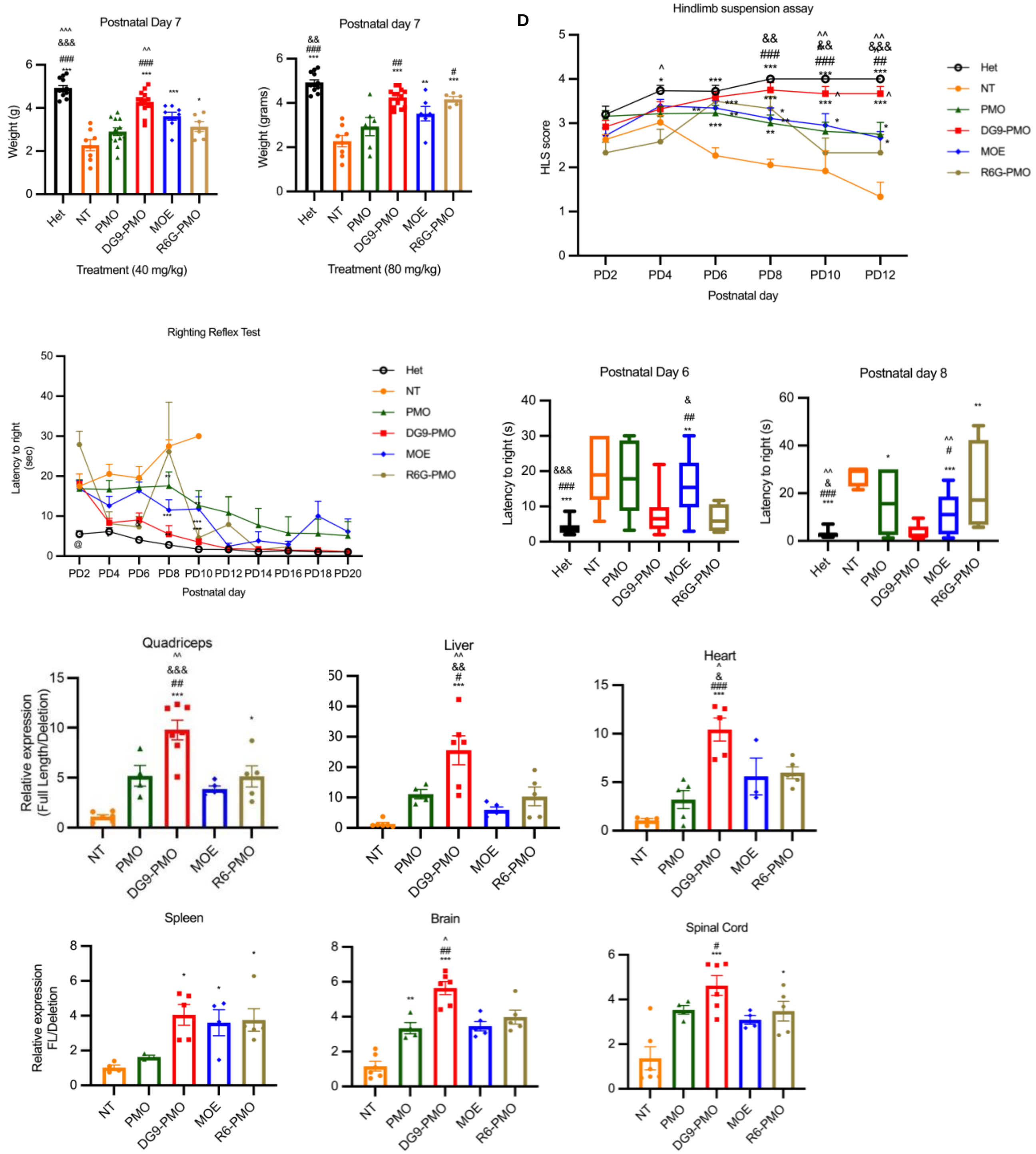

Figure 1. Improved efficacy following DG9-PMO treatment compared to R6-PMO in SMA mice. (**A**) Survival curves of heterozygous mice (Het), non-treated (NT), unconjugated-PMO (PMO), DG9-PMO, MOE and R6G-PMO injected at PD0 at a dose of 40 mg/kg. n=15 (Hets), n=22 (NT), n=49 (unconjugated PMO), n=14 (DG9-PMO), n=29 (MOE), and n=6 (R6G-PMO), ($p \leq 0.0001$, log-rank Mantel Cox test). (**B**) A representative image of heterozygous mouse, mice treated with either 80 mg/kg or 40 mg/kg of DG9-PMO, mouse injected with unconjugated- PMO (40 mg/kg), and saline-treated NT mouse at PD7 (left-to-right). (**C**) Weight of mice at PD7 administered with either 40 or 80 mg/kg doses. Each dot (symbol) indicates a neonatal pup. (**D**) Hindlimb suspension assay (HLS). Mice were treated with 40 mg/kg AOs at PD0. Scored is based on the position of the hindlimbs when suspended from a falcon tube. (**E**) Righting reflex test. Mice were treated with 40 mg/kg AOs at PD0. The ability of mice to right themselves on their paws was measured every alternate day between PD2 to PD20 (left). The mean righting reflex time at PD6 and PD8 was also indicated (right: box whiskers plots). Box edges, 25th and 75th percentiles; central line, median; whiskers, range. (**F**) Relative expression levels of full length *SMN2* (*FL-SMN2*) compared to deleted *SMN2* transcripts *(Δ7 SMN2*) measured by qPCR following 40 mg/kg ASO or PBS treatments. In **C**, **E (**box whisker plots**)**, and **F**, One-way ANOVA followed by post hoc Tukey's test was performed. *p<0.05, **p<0.01, ***p<0.005. In **D** and **E**, Two-way ANOVA followed by Sidak's multiple comparison was performed. *p<0.03; **p<0.002; ***p<0.0002.*NT, #PMO, @DG9-PMO, &MOE, ^R6-PMO. Error bars: SEM

Figure 2.

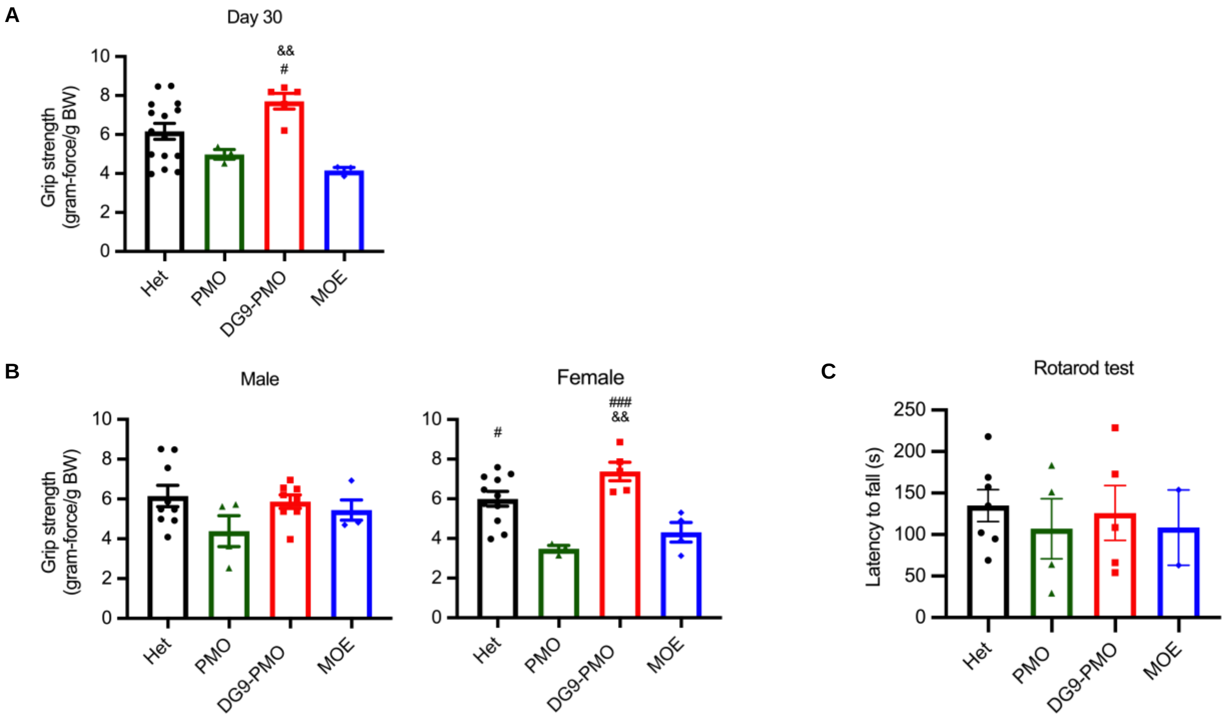

Figure 2. Muscle strength and motor function tests in DG9-PMO treated adult SMA mice. (**A**) Forelimb grip strength normalized to the body weight measured at PD30. 80 mg/kg of AOs were injected at PD0. (**B**) Forelimb grip strength measured in adult males and females at PD30. 40 mg/kg of AOs were injected at PD0. (**C**) Rotarod test with an acceleration profile. Measured at PD 30-35. Each mouse was subjected to three trials spaced 20 minutes from one other. The maximum time on the beam was noted down. 40 mg/kg of AOs were injected at PD0. One-way ANOVA followed by post hoc Tukey's test was performed *NT, #PMO, @DG9-PMO, &MOE. *p<0.05, **p<0.01, ***p<0.005. Error bars: SEM.

Figure 3.

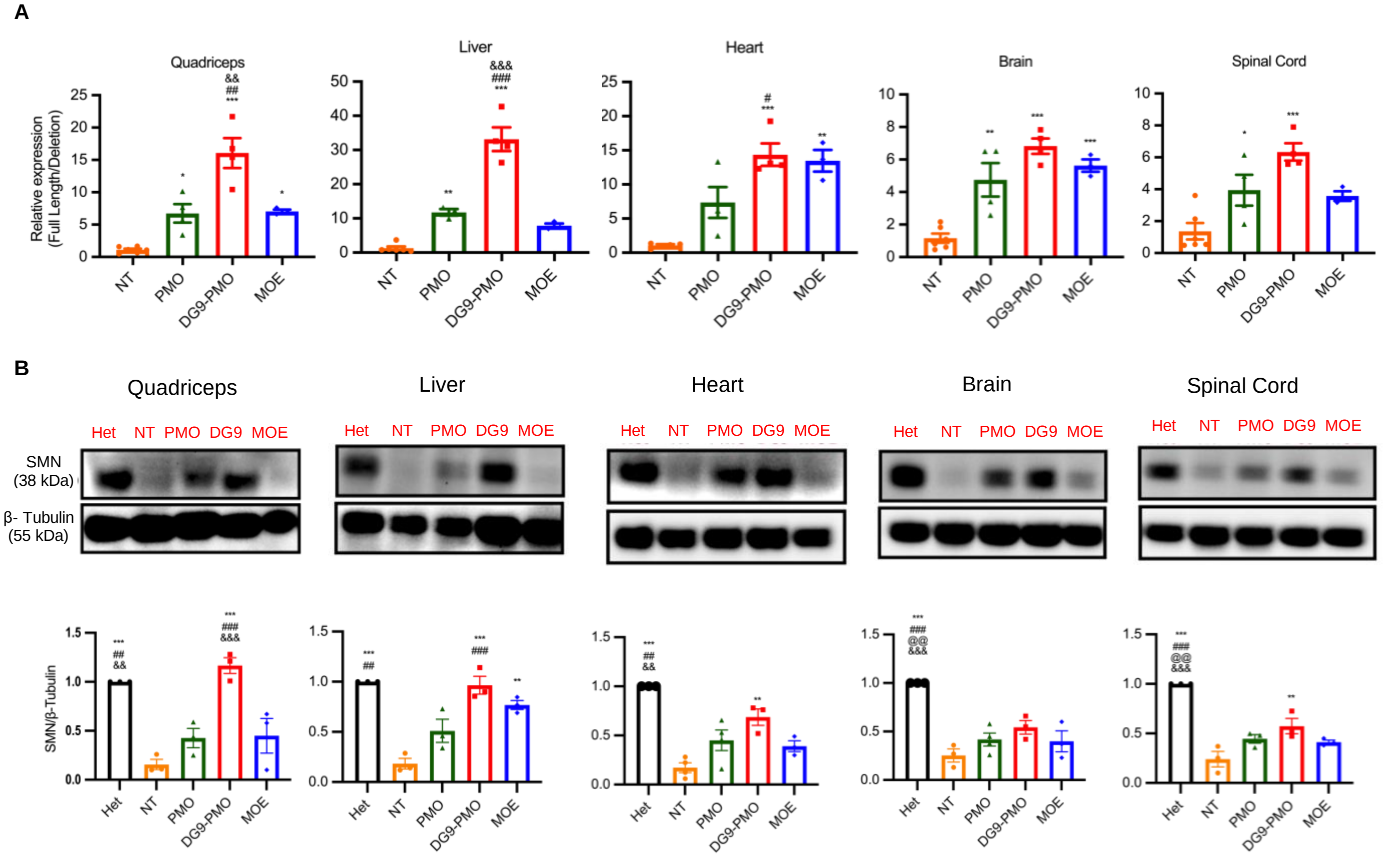

Figure 3. DG9-PMO treatment induces SMN expression in a dose-dependent manner in SMA mice. (**A**) Relative expression levels of full length *SMN2* (*FL-SMN2*) compared to deleted *SMN2* transcripts *(Δ7 SMN2*) measured by qPCR in NT and treated mice in the quadriceps muscle, liver, heart, brain, and spinal cord following 80 mg/kg AO treatment. (**B**) Representative images from western blotting and the quantification of SMN levels relative to β- Tubulin. The heterozygous mice were used as a control relative SMN expression set to 1. The tissues from the 80 mg/kg treated mice were collected at PD7. In **A** and **B**, one-way ANOVA followed by post hoc Tukey's test was performed *NT, #PMO, @DG9-PMO, &MOE. *p<0.05, **p<0.01, ***p<0.005. Error bars: SEM.

Figure 4.

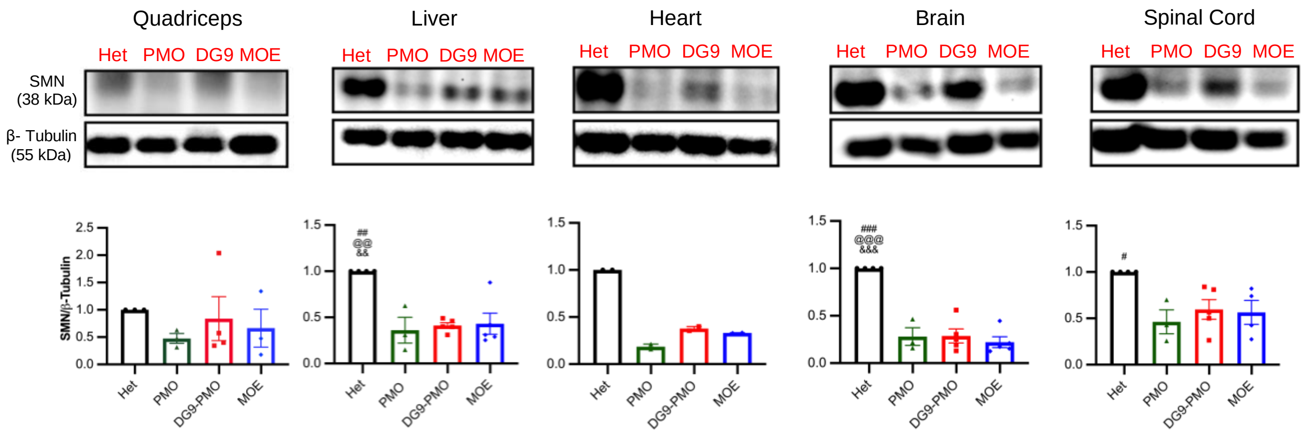

Figure 4. DG9-PMO maintains SMN levels in SMA mice at postnatal day 30. Representative images from western blotting and the quantification of SMN levels. The tissues were collected at PD30 from 40 mg/kg groups, relative to β- Tubulin. The heterozygous mice were used as a control with the relative SMN expression set to 1. Statistics performed using one-way ANOVA followed by post hoc Tukey's test. #PMO, @DG9-PMO, &MOE. *$p<0.05$, **$p<0.01$, ***$p<0.005$. Error bars: SEM.

Figure 5.

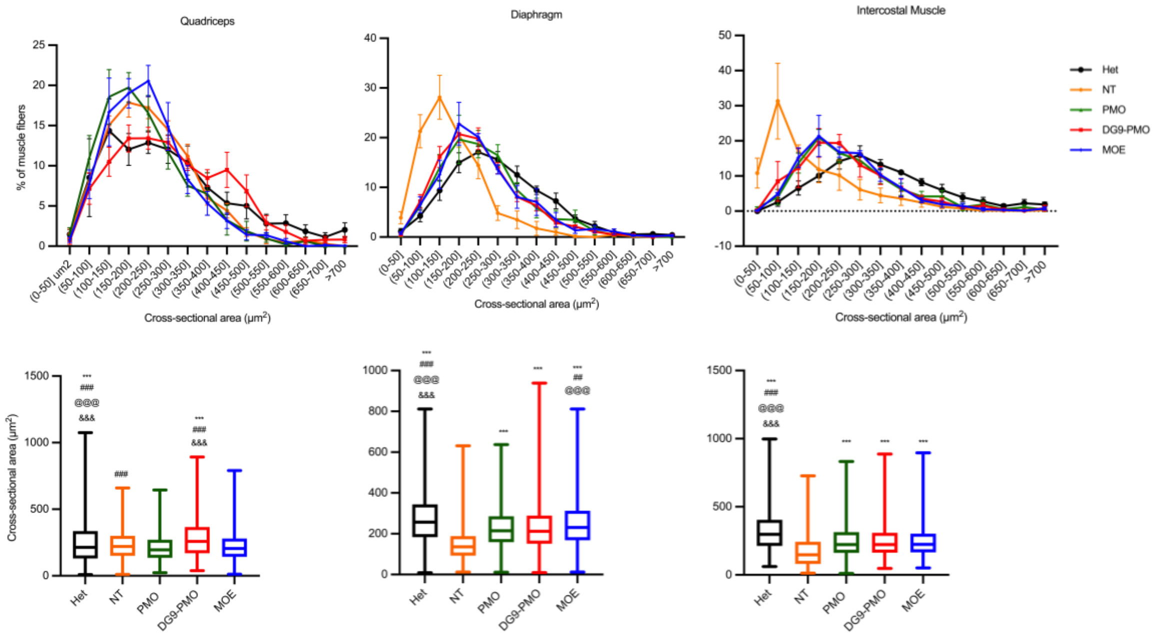

Figure 5. DG9-PMO improves the muscle pathology in SMA mice at postnatal day 7. Frequency distribution of cross-sectional area (CSA) (μm^2) and the quantification of individual myofibers from the quadriceps muscle, diaphragm and intercostal muscle harvested at PD7. Box edges, 25th and 75th percentiles; central line, median; whiskers, range (n=3-7 per group), with around 1292-1653 fibers for the quadriceps, 917-1746 fibers for the diaphragm and 642-1127 for the intercostal muscle. 40 mg/kg of AOs were injected at PD0. Statistics was performed using one-way ANOVA followed by post hoc Tukey's test. *NT, #PMO, @DG9-PMO, &MOE. *p<0.05, **p<0.01, ***p<0.005. Error bars: SEM.

Figure 6.

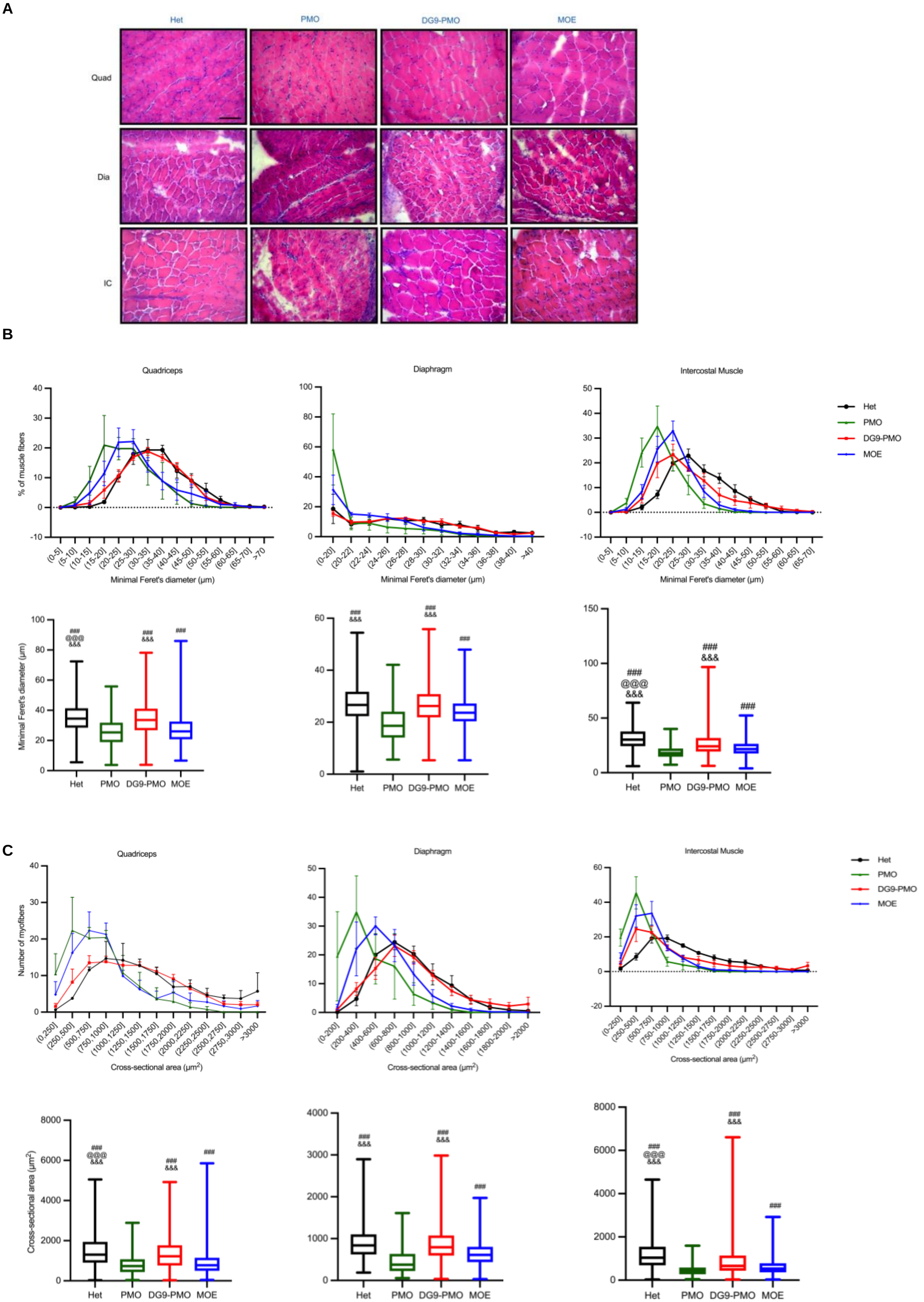
A
Het
PMO
DG9-PMO
MOE
Quad
Dia
IC
B
Quadriceps
Diaphragm
Intercostal Muscle
% of muscle fibers
Minimal Feret's diameter (μm)
Het
PMO
DG9-PMO
MOE
Minimal Feret's diameter (μm)
C
Quadriceps
Diaphragm
Intercostal Muscle
Number of myofibers
Cross-sectional area (μm²)
Cross-sectional area (μm²)

Figure 6. DG9-PMO improves the muscle pathology in adult SMA mice at postnatal day 30. (**A**) Representative images from H & E staining of the quadriceps muscle (top row), diaphragm (middle row) and the intercostal muscle (bottom row) at PD30 in the heterozygous, NT control and treated groups. 40 mg/kg of AOs were injected at PD0. Scale bar: 100 µm. (**B**) Frequency distribution of the minimal Feret's diameter (µm) and the quantification of individual myofibers from tissues collected at PD30 shown below. (**C**) Frequency distribution of cross-sectional area (CSA) (μm^2) and the quantification of individual myofibers shown below. Box edges, 25th and 75th percentiles; central line, median; whiskers, range (n=3-6 per group), with around 960-1868 fibers for the quadriceps, 983-1436 fibers for the diaphragm and 800-1802 for the intercostal muscle. 40 mg/kg of AOs were injected at PD0. Statistics was performed using one-way ANOVA followed by post hoc Tukey's test. #PMO, @DG9-PMO, &MOE. *p<0.05, **p<0.01, ***p<0.005. Error bars: SEM.

Figure 7.

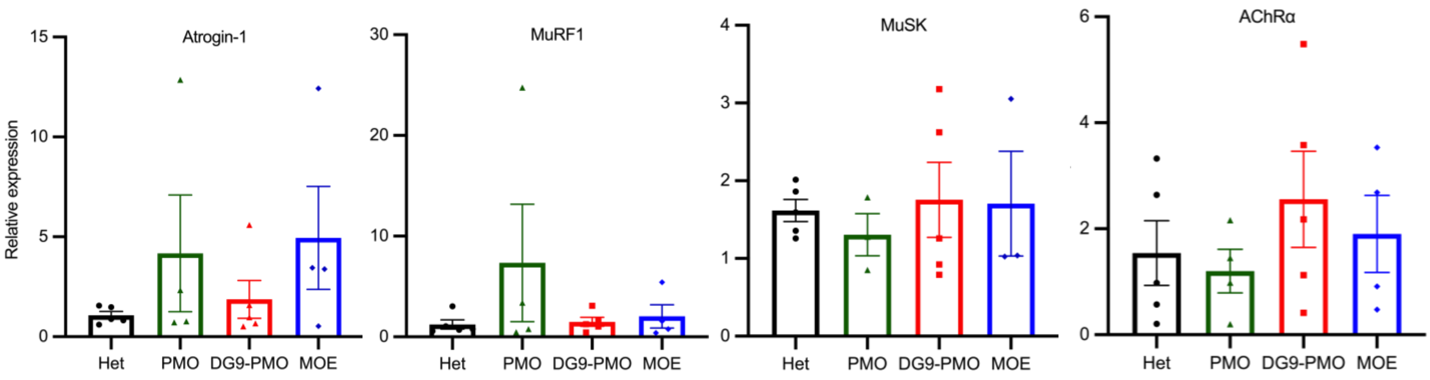

Figure 7. Evaluation of the expression of denervation markers at PD30. Expression of denervation markers Atrogin-1 and MuRF-1 as well as markers MuSK and AChRα necessary for NMJ maintenance relative to 18S ribosomal RNA in the quadriceps muscle at PD30 following treatment. N=4-5 per group with both males and females included. One-way ANOVA followed by post hoc Tukey's test was performed.*p<0.05, **p<0.01. Error bars: SEM.

Figure 8.

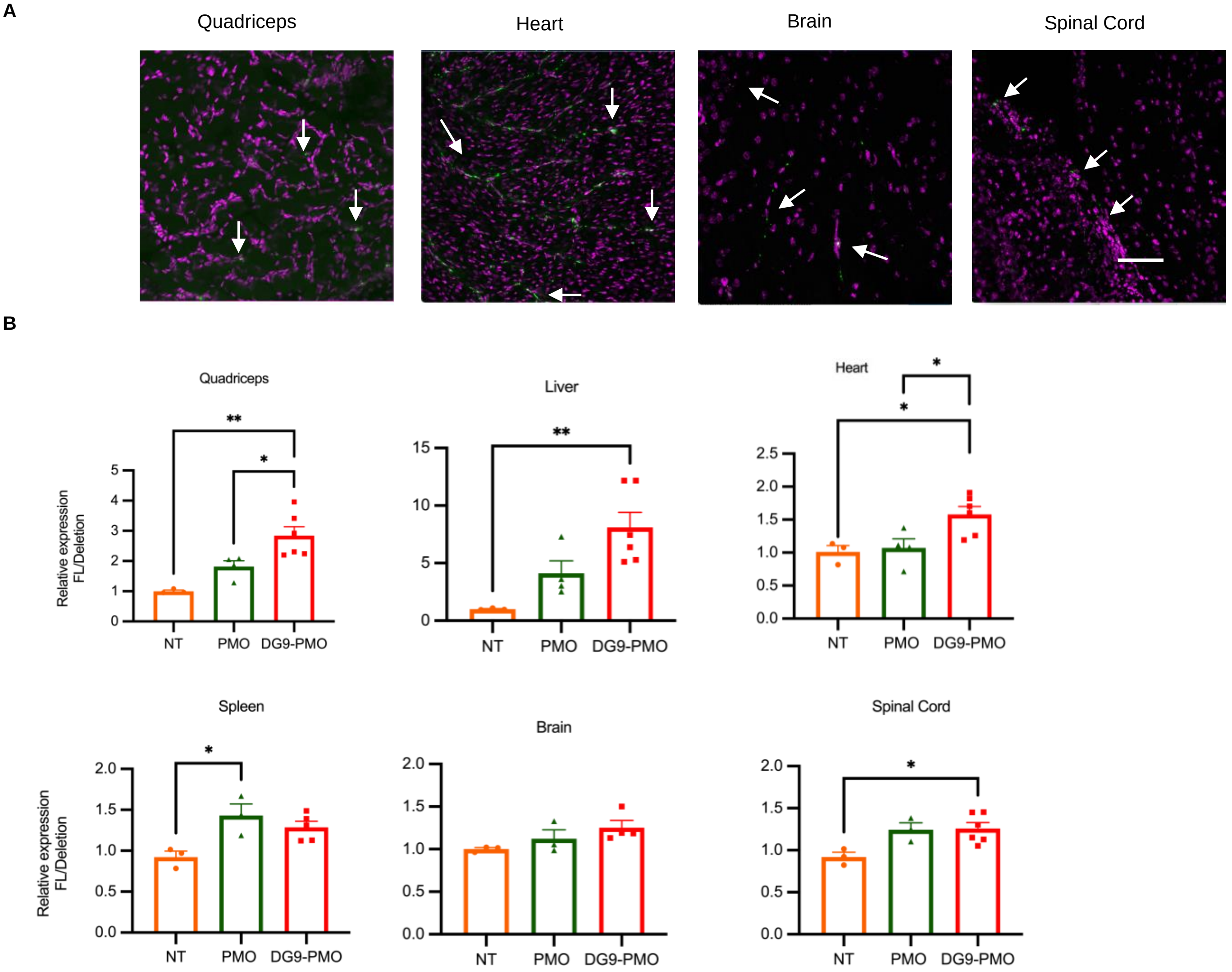

Figure 8. DG9-PMO bypasses the blood-brain barrier in a mild SMA model. (**A**) Representative immunohistochemistry images at PD13 from the quadriceps muscle, heart, brain, and the spinal cord, following fluorescently tagged DG9-PMO (green) subcutaneous administration at PD5 in F0 mice (*Smn*$^{-/-}$ *SMN2*$^{+/+}$). Magenta: DAPI. n=3 per group. White arrows indicate DG9-PMO overlapped with nuclei (DAPI). Scale bar: 50 µm. (**B**) Relative expression levels of full length *SMN2* (*FL-SMN2*) compared to deleted *SMN2* transcripts (Δ*7 SMN2*) in the quadriceps muscle, liver, heart, spleen, brain, and spinal cord. Saline, unconjugated- PMO, and DG9-PMO were injected into PD5 mice subcutaneously (n=3-6 per group). The tissues were collected at PD7. One-way ANOVA followed by post hoc Tukey's test was performed. *p<0.05, **p<0.01, ***p<0.005. Error bars: SEM.

Figure 9.

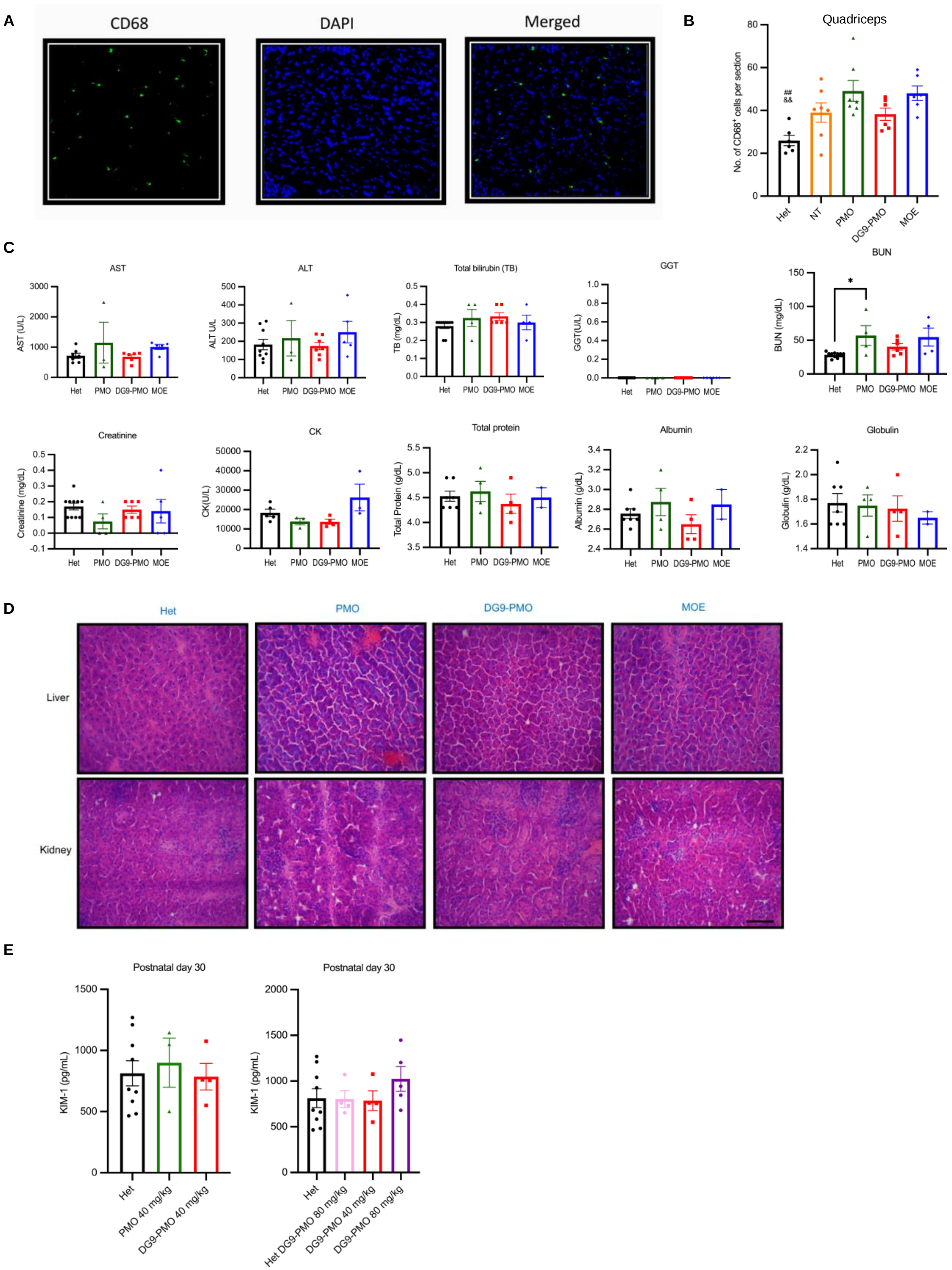
A
CD68
DAPI
Merged
B
Quadriceps
No. of CD68+ cells per section
##
&&
Het
NT
PMO
DG9-PMO
MOE
C
AST
AST (U/L)
ALT
ALT U/L
Total bilirubin (TB)
TB (mg/dL)
GGT
GGT(U/L)
BUN
BUN (mg/dL)
*
Creatinine
Creatinine (mg/dL)
CK
CK(U/L)
Total protein
Total Protein (g/dL)
Albumin
Albumin (g/dL)
Globulin
Globulin (g/dL)
D
Het
PMO
DG9-PMO
MOE
Liver
Kidney
E
Postnatal day 30
KIM-1 (pg/mL)
Het
PMO 40 mg/kg
DG9-PMO 40 mg/kg
Postnatal day 30
KIM-1 (pg/mL)
Het
Het DG9-PMO 80 mg/kg
DG9-PMO 40 mg/kg
DG9-PMO 80 mg/kg

Figure 9. DG9-PMO treatment does not lead to an apparent immune response and toxicity. (**A**) Representative images from immunostaining of CD68$^+$ macrophages (green) and DAPI (blue) in the quadriceps muscle collected at PD7. (**B**) Average CD68$^+$ cells per section. 3-5 sections from the quadriceps muscle from each mouse were used for analysis. (n=3-5 per group). scale bar: 100 µm. (**C**) Serum analysis for ALP, ALT, AST, total bilirubin, GGT, BUN, creatinine, CK, total protein, albumin, and globulin. Serum was collected at PD30. (n=2-7 per group). (**D**) Representative images from H and E staining of the liver (top row) and kidney (bottom row) at PD30. No apparent morphological differences were observed between the healthy heterozygous control and treated groups. scale bar: 100 µm. 40 mg/kg of AOs were injected at PD0 in **A-D**. (**E**) Urinary KIM-1 analysis from mice treated with 40 or 80 mg/kg of DG9-PMO (DG9-PMO 40, DG9-PMO 80) or 40 mg/kg of unconjugated PMO (PMO 40) at PD30. Heterozygous healthy control mice (Het) treated with PBS alone (Het) or DG9-PMO 80 mg/kg (Het DG9-PMO 80). One-way ANOVA followed by post hoc Tukey's test was performed. *$p<0.05$, **$p<0.01$, ***$p<0.005$. Error bars: SEM

Supplemental Tables

Table 1: List of primers used in this study.

Target	Sequence (5'-3')
Smn-S1	ATAACACCACCACTCTTACTC
Smn-S2	GTAGCCGTGATGCCATTGTCA
Smn-H1	AGCCTGAAGAACGAGATCAGC
FL-SMN2	F: GCTATCATACTGGCTATTATATGGGTTTT<br>R: CTCTATGCCAGCATTTCTCCTTAAT
$\Delta SMN2$	F: TCTGGACCACCAATAATTCCCC<br>R: ATGCCAGCATTTCCATATAATAGCC
mGAPDH	F: GAGAAACCTGCCAAGTA<br>R: CAGTGTAGCCCAAGATG
Atrogin-1 (50)	F: GCAGAGAGTCGGCAAGTC<br>R: CAGGTCGGTGATCGTGAG
MuRF-1 (50)	F: AGTGTCCATGTCTGGAGGTCGTTT<br>R: ACTGGAGCACTCCTGCTTGTAGAT
MuSK (49)	F: GCCTTCAGCGGGACTGAG<br>R: GAGGCGTGGTGACAGG
AChRα (49)	F: GCCATTAACCCGGAAAGTGAC<br>R: CCCCGCTCTCCATGAAGTT
18S (50)	F: GGGGAGTATGGTTGCAAAGC<br>R: CGCTCCACCAACTAAGAACG

Unedited and uncut blots/gels

Full unedited gel for Figure 2B (Quadriceps)

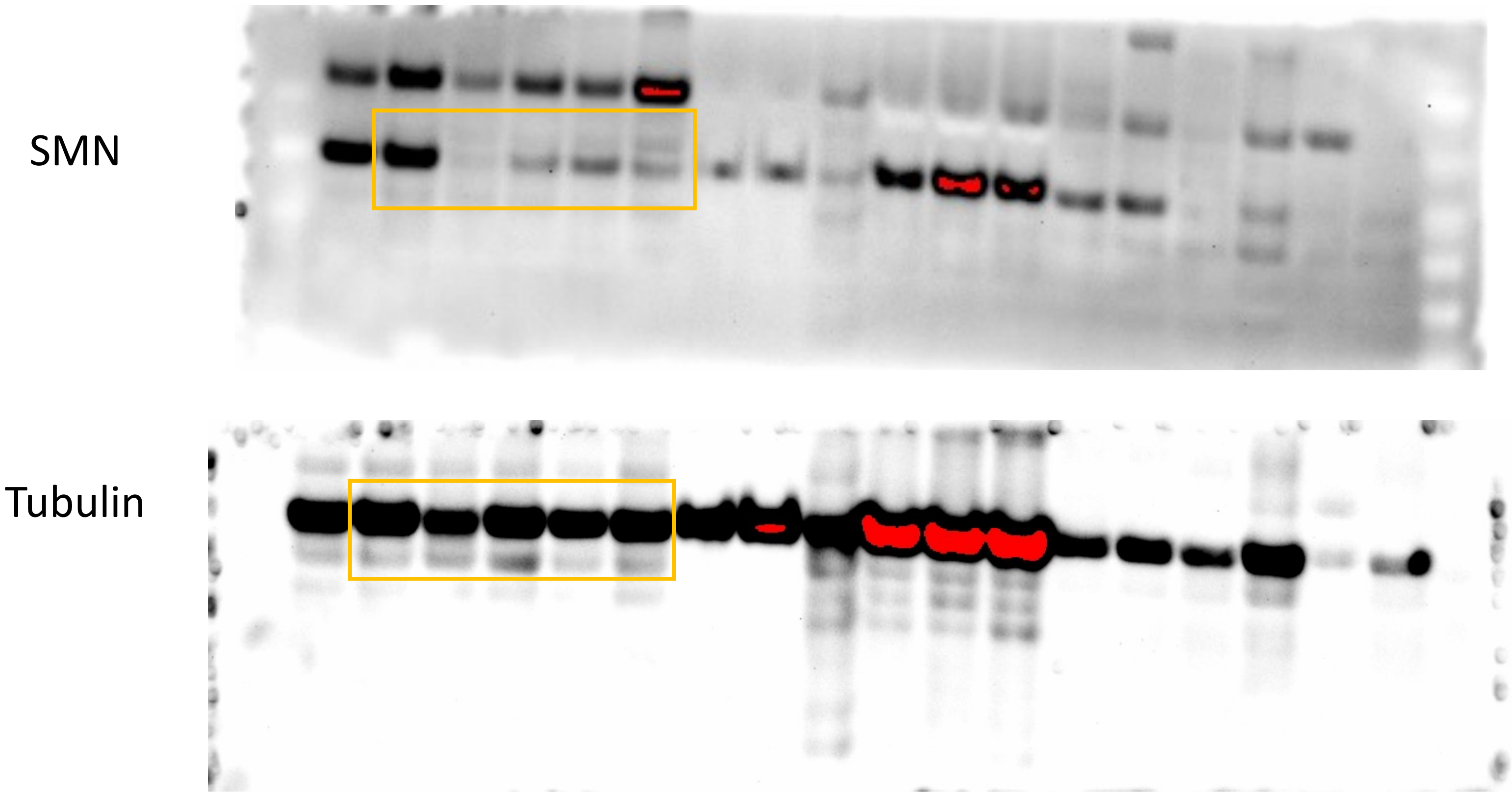

Full unedited gel for Figure 2B (Liver)

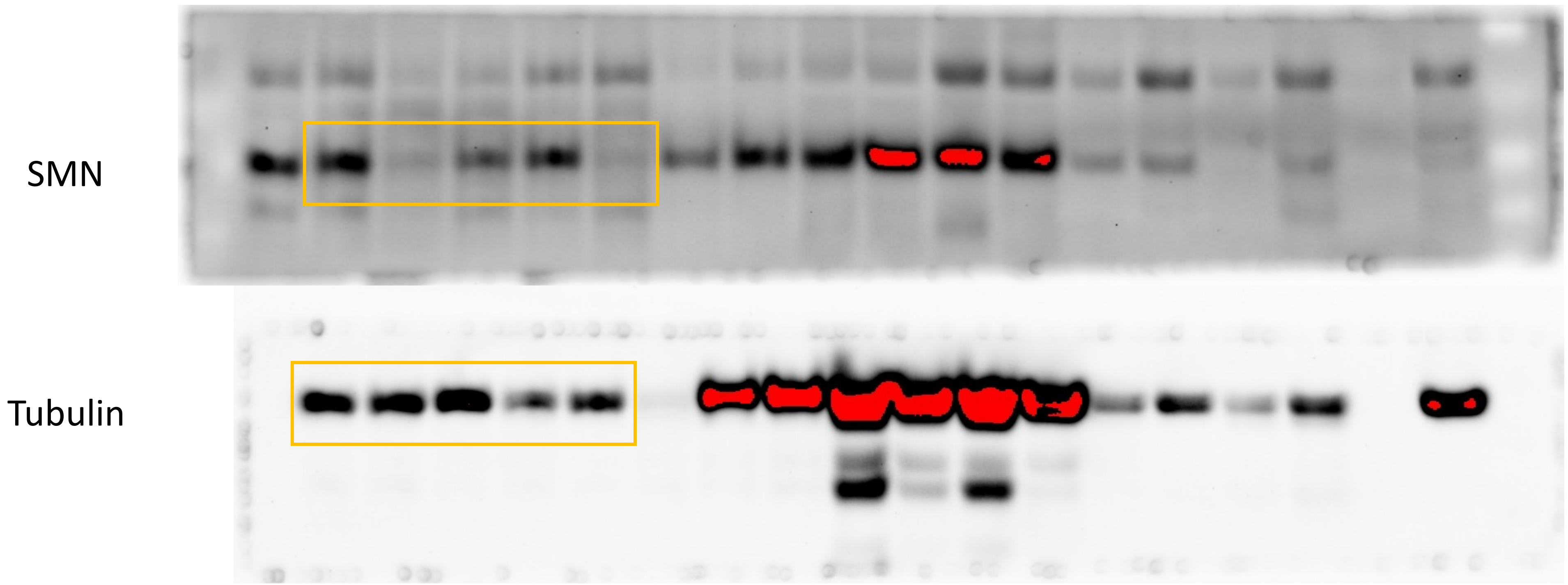

Full unedited gel for Figure 2B (Heart)

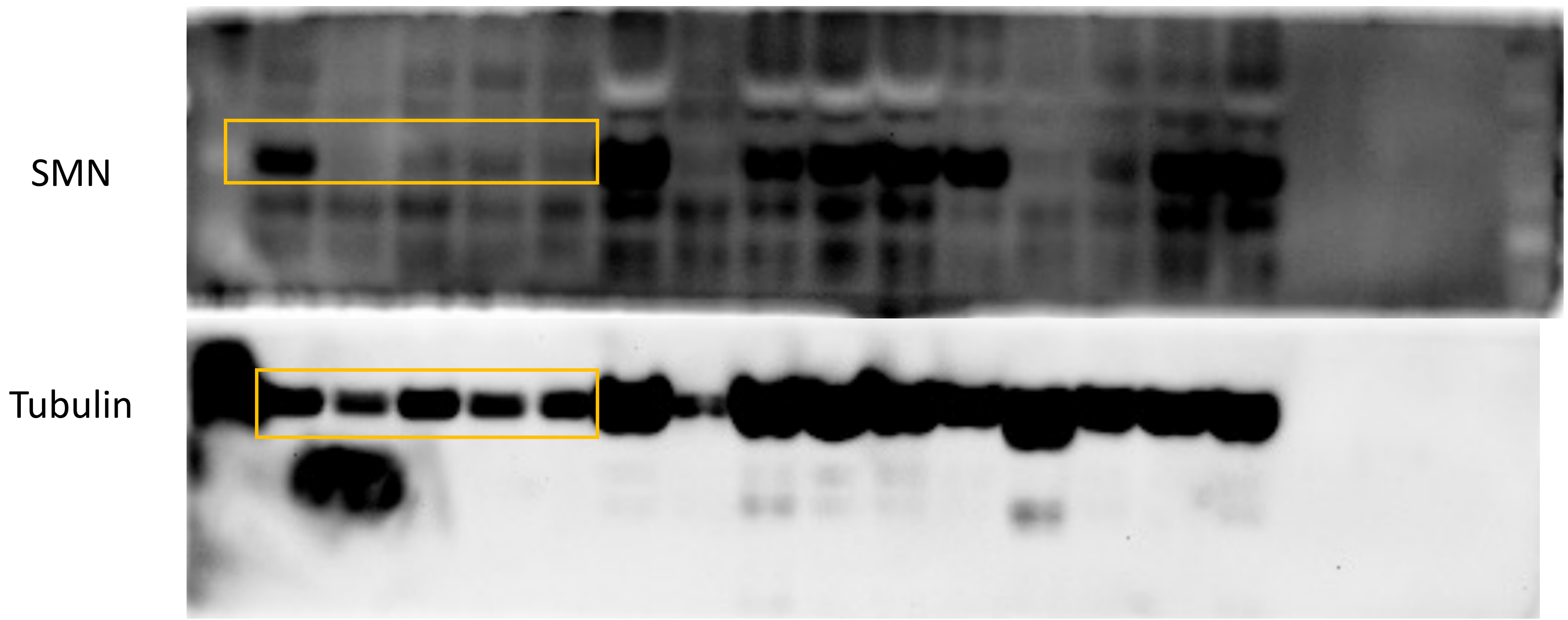

Full unedited gel for Figure 2B (Brain)

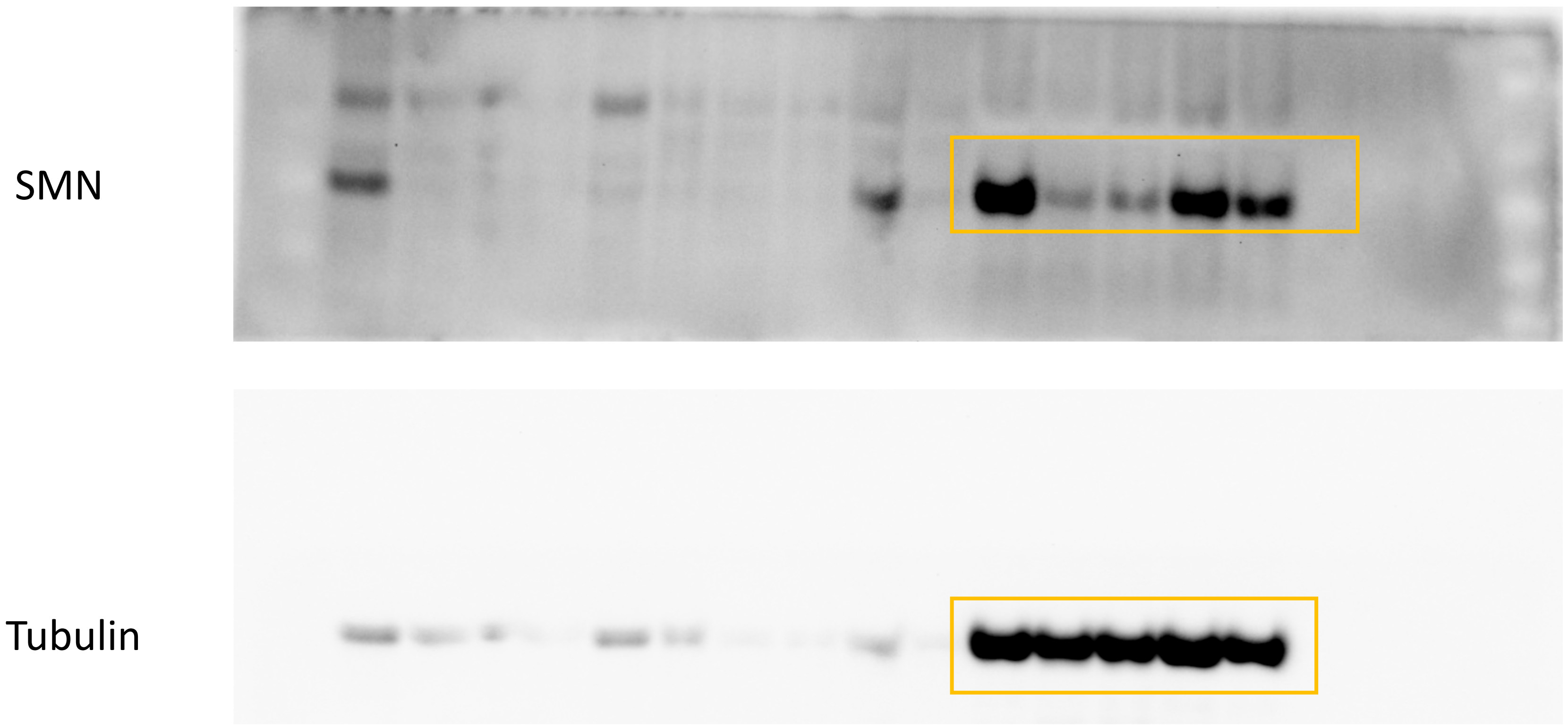

Full unedited gel for Figure 2B (Spinal Cord)

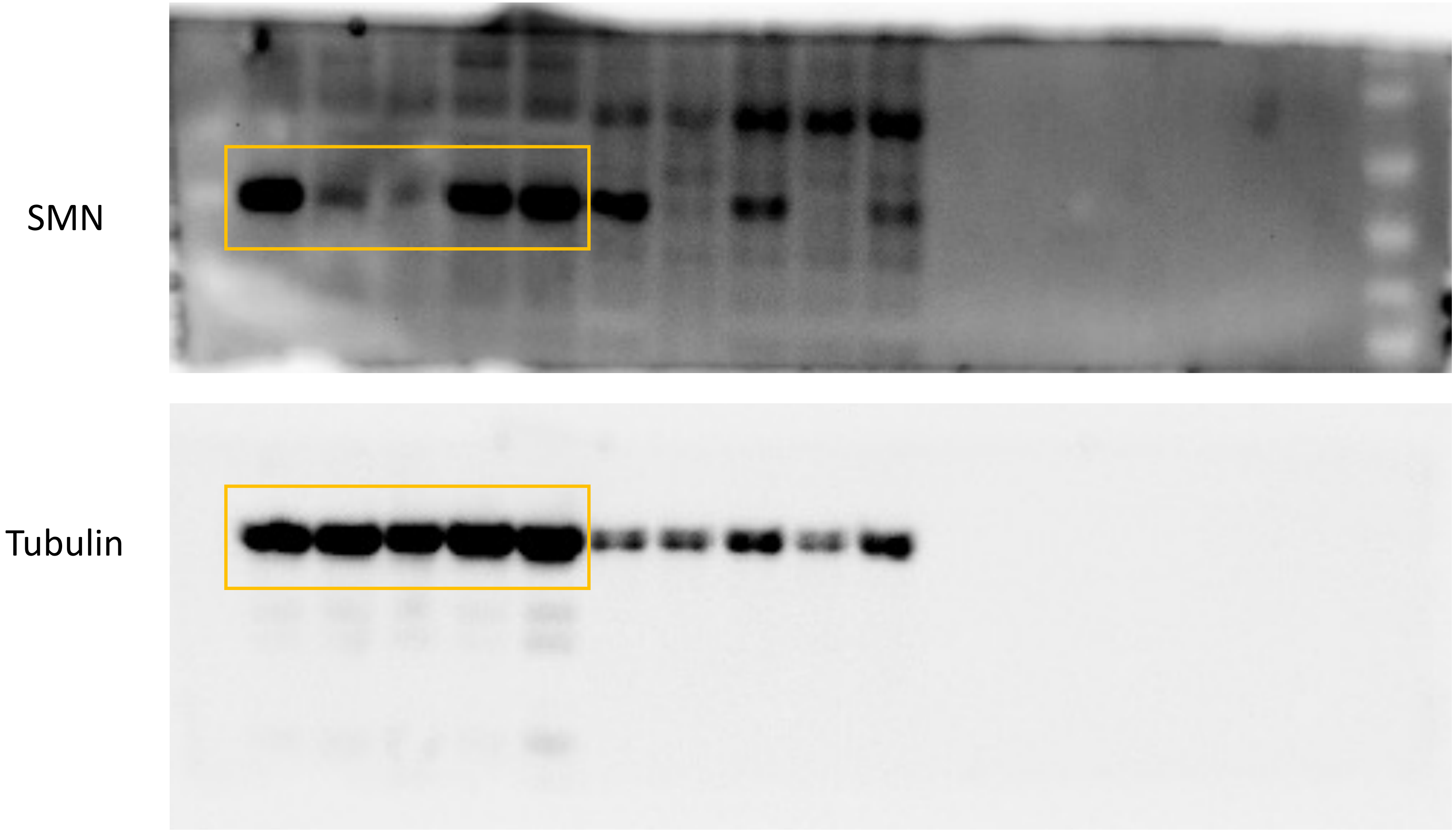

Full unedited gel for Supplemental Figure 3B (Quadriceps)

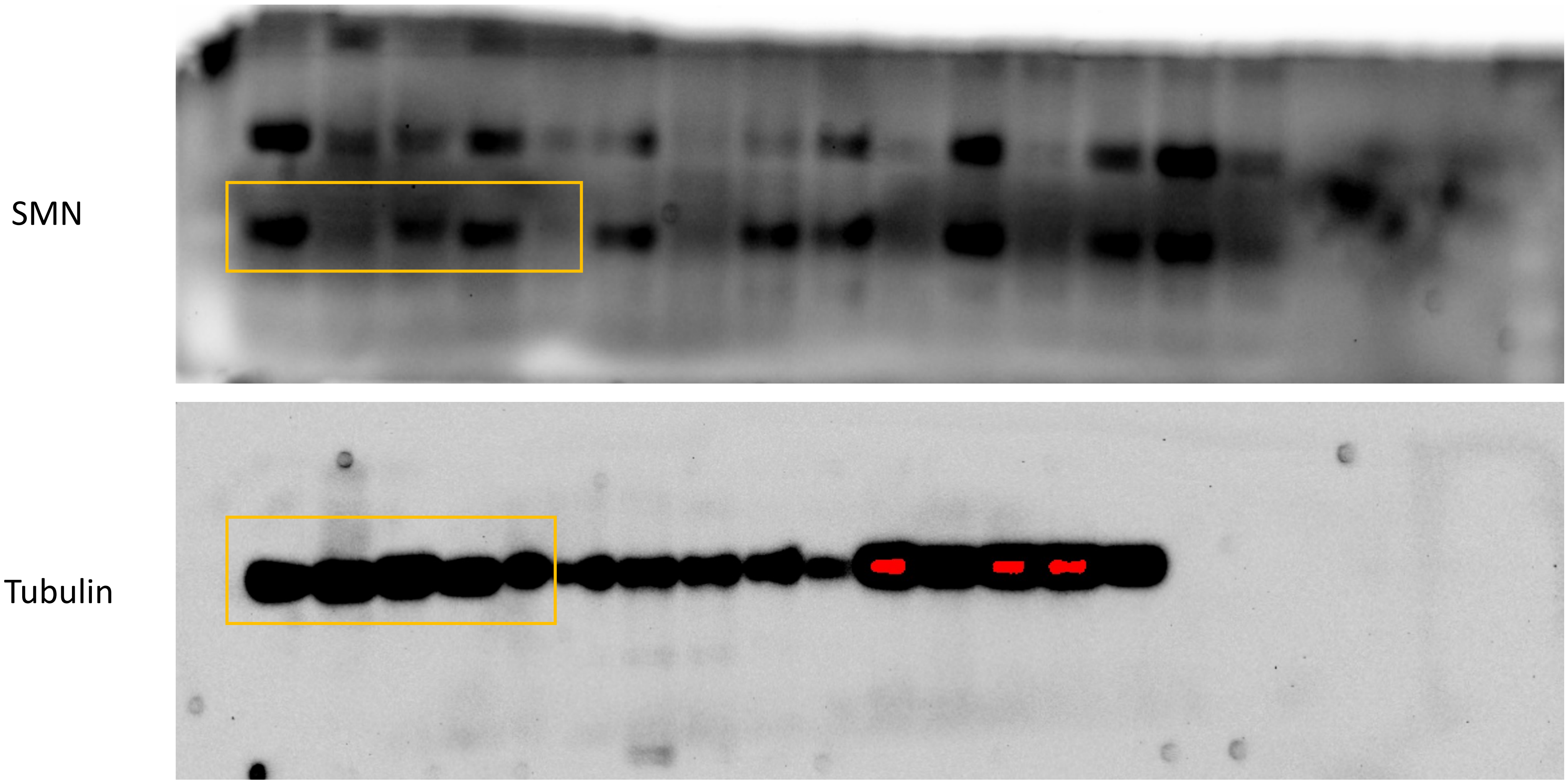

Full unedited gel for Supplemental Figure 3B (Liver)

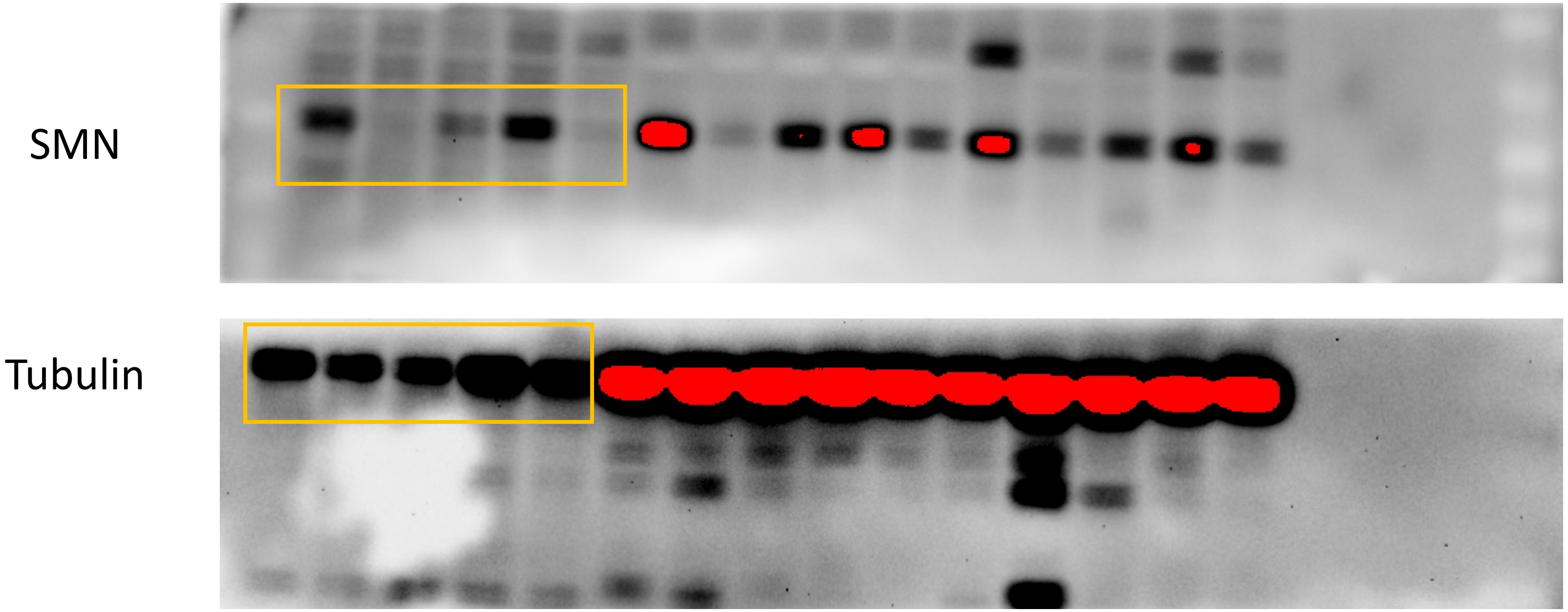

Full unedited gel for Supplemental Figure 3B (Heart)

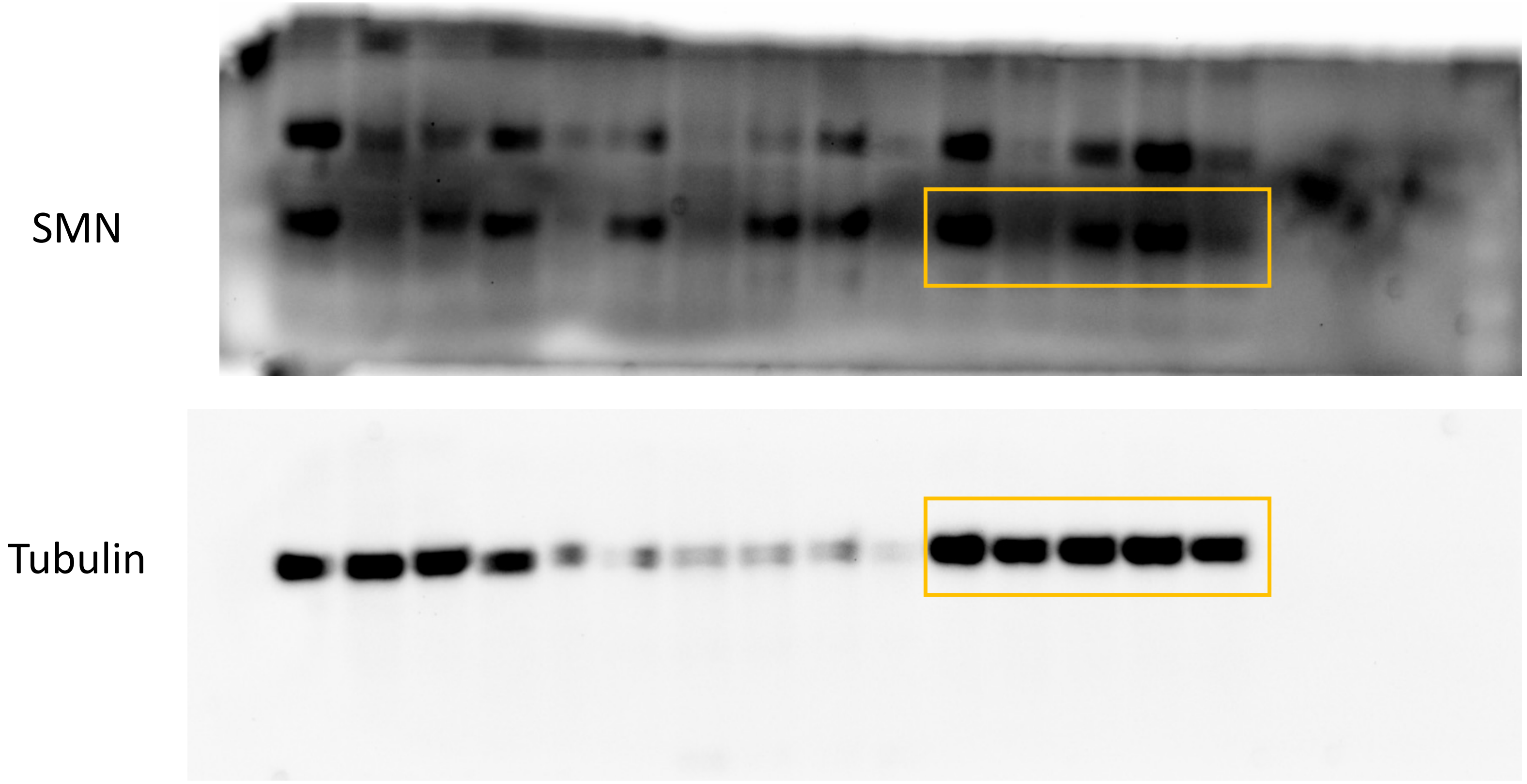

Full unedited gel for Supplemental Figure 3B (Brain)

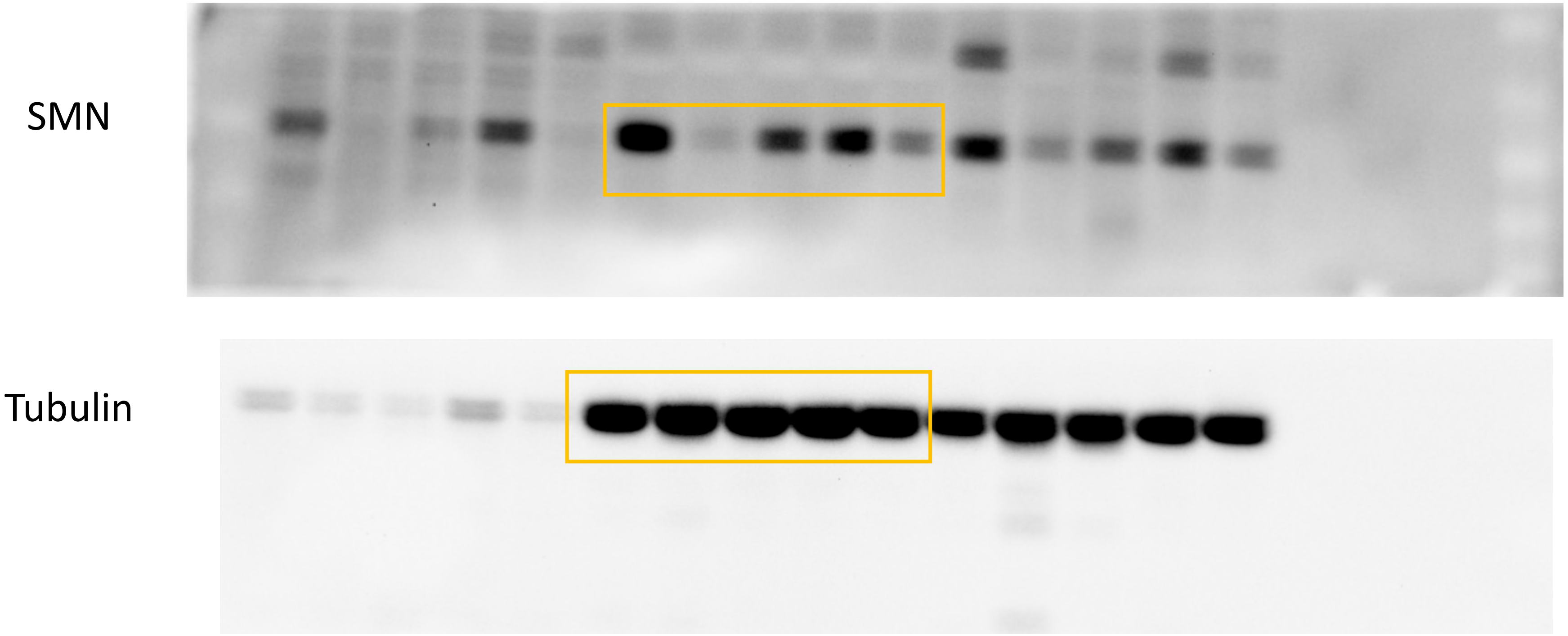

Full unedited gel for Supplemental Figure 3B (Spinal Cord)

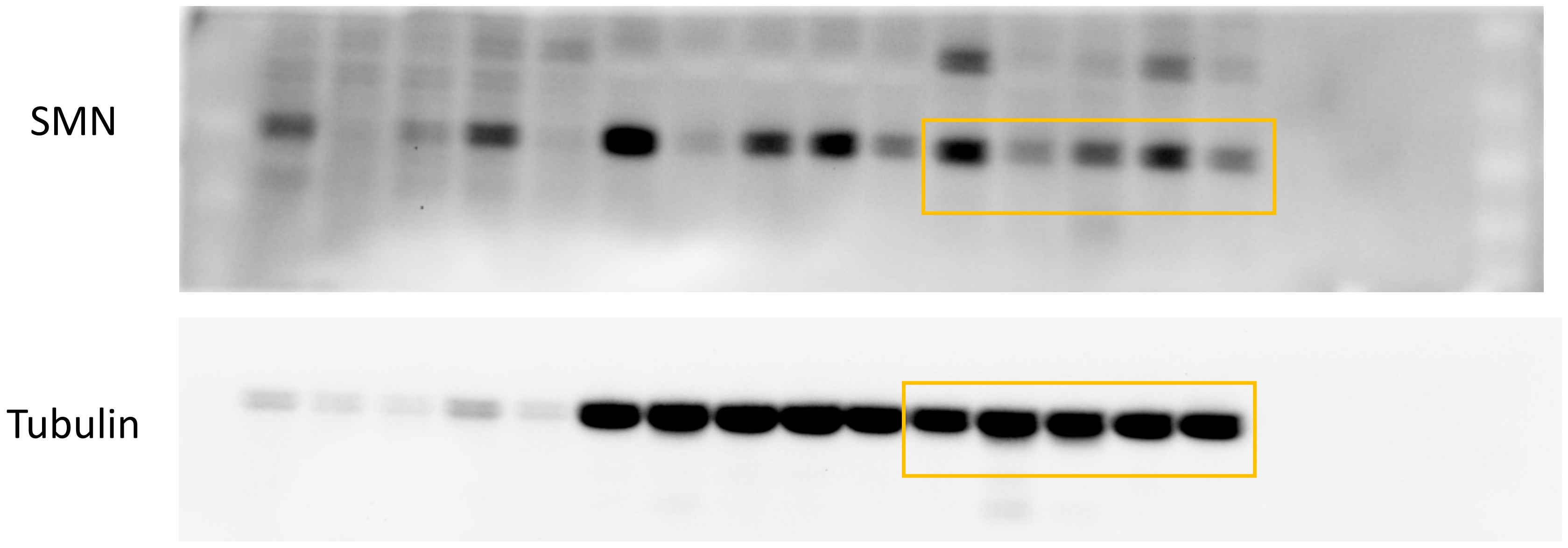

Full unedited gel for Supplemental Figure 4 (Quadriceps)

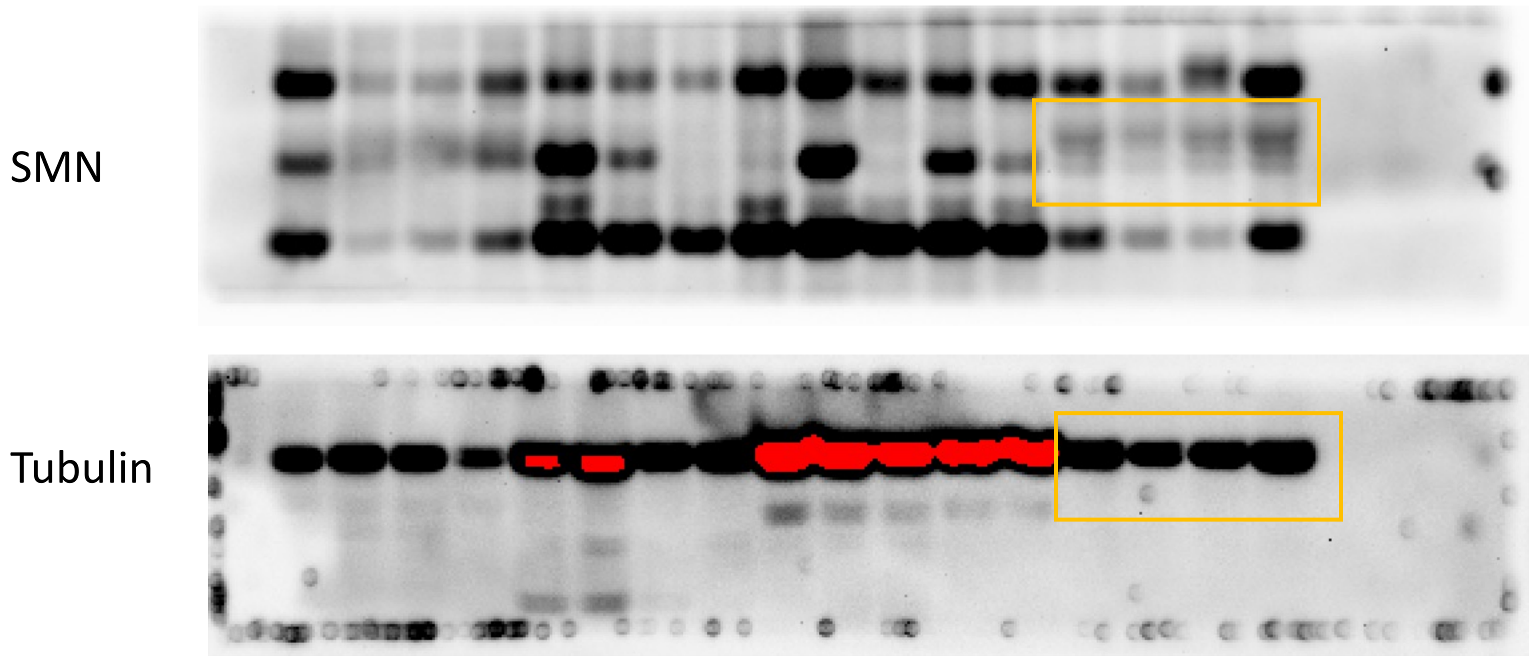

Full unedited gel for Supplemental Figure 4 (Liver)

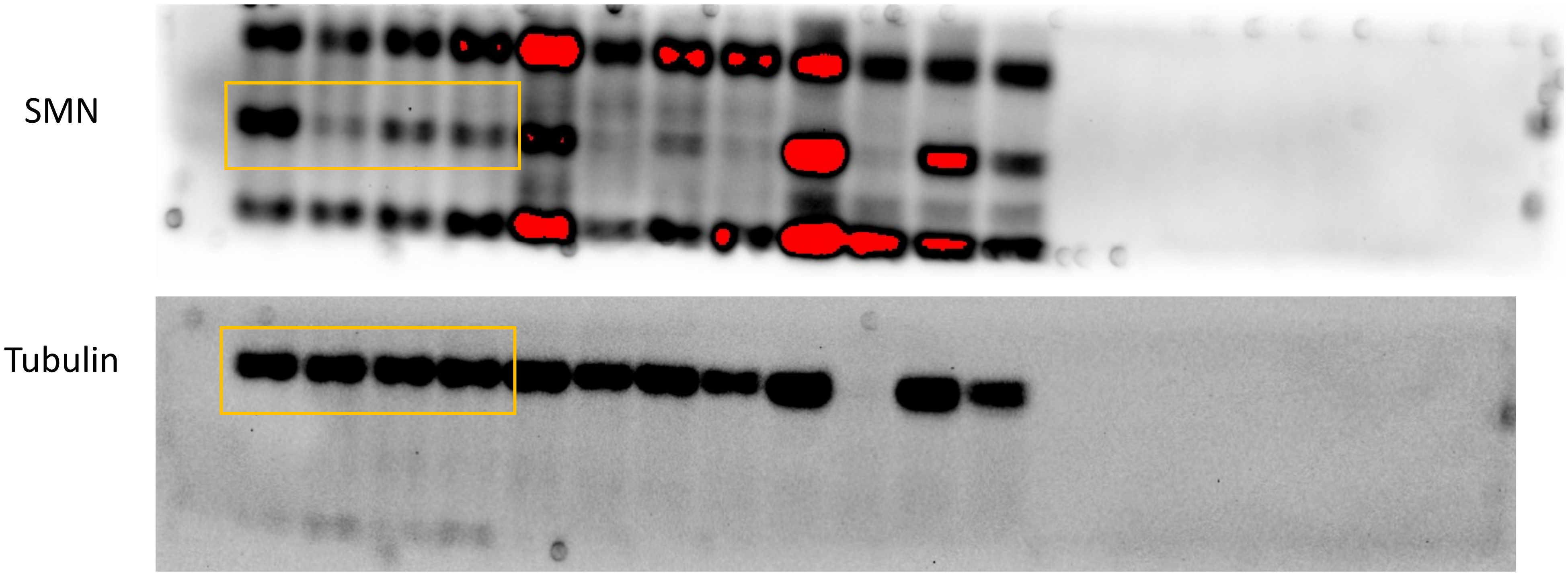

Full unedited gel for Supplemental Figure 4 (Heart)

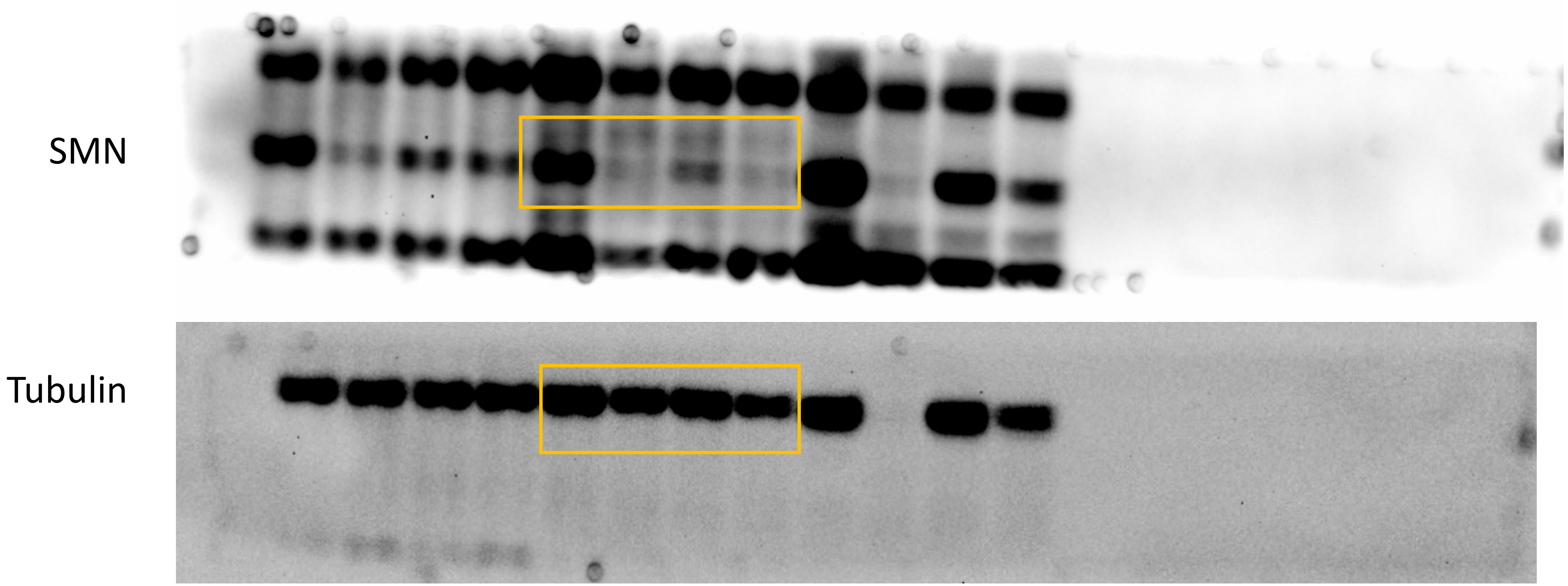

Full unedited gel for Supplemental Figure 4 (Brain)

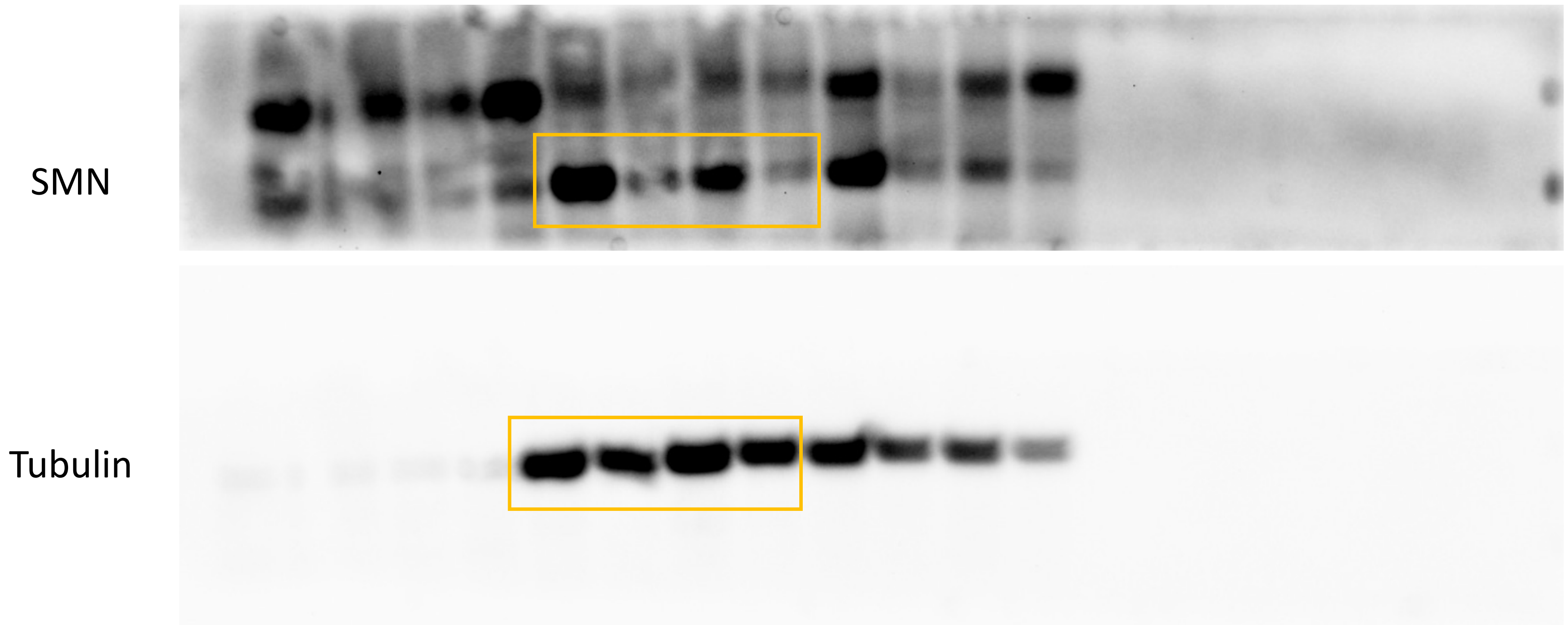

Full unedited gel for Supplemental Figure 4 (Spinal Cord)

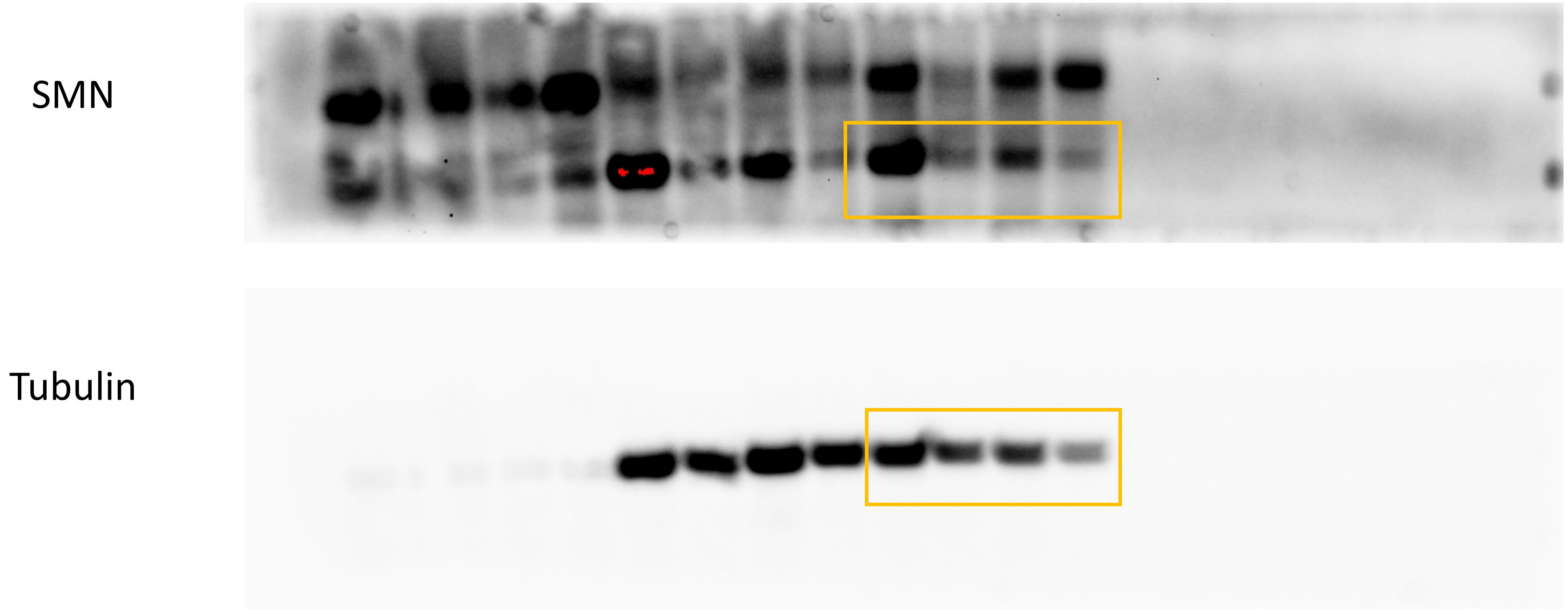

Supplement: Supplemental data [file jciinsight-8-160516-s232.pdf]
